# Quantifying the effect of sagittal plane joint angle variability on bipedal fall risk

**DOI:** 10.1371/journal.pone.0262749

**Published:** 2022-01-26

**Authors:** Amy Mitchell, Anne E. Martin

**Affiliations:** Department of Mechanical Engineering, The Pennsylvania State University, University Park, PA, United States of America; University of Innsbruck, AUSTRIA

## Abstract

Falls are a major issue for bipeds. For elderly adults, falls can have a negative impact on their quality of life and lead to increased medical costs. Fortunately, interventional methods are effective at reducing falls assuming they are prescribed. For biped robots, falls prevent them from completing required tasks. Thus, it is important to understand what aspects of gait increase fall risk. Gait variability may be associated with increased fall risk; however, previous studies have not investigated the variation in the movement of the legs. The purpose of this study was to determine the effect of joint angle variability on falling to determine which component(s) of variability were statistically significant. In order to investigate joint angle variability, a physics-based simulation model that captured joint angle variability as a function of time through Fourier series was used. This allowed the magnitude, the frequency mean, and the frequency standard deviation of the variability to be altered. For the values tested, results indicated that the magnitude of the variability had the most significant impact on falling, and specifically that the stance knee flexion variability magnitude was the most significant factor. This suggests that increasing the joint variability magnitude may increase fall risk, particularly if the controller is not able to actively compensate. Altering the variability frequency had little to no effect on falling.

## Introduction

Falls in bipeds are widely acknowledged to be undesirable, but difficult to predict [1–4]. For elderly adults, falls can cause death, reduce quality of life, and increase medical costs [5]. Exercise and risk management can reduce falls [6–8], motivating identification of potential fallers to provide interventional care. While falls are not as catastrophic for robotic bipeds, falling prevents completion of required tasks and potentially damages the robot. Although humans and robots are certainly different, their walking dynamics share many similarities [9–12], allowing robotic modeling and control methods to be used to investigate fall risk for both humans and robots.

One challenge with predicting falls is that they have many potential causes. Robotic studies have primarily evaluated falls due external factors, such as walking across uneven terrain [13] or due to velocity disturbances such as a push [14, 15]. These works also tend to focus on discrete, rather than continuous, perturbations. In contrast, human studies generally focus on finding correlations between risk factors [16–19] or gait parameters [20–22] and fall risk. Another source of falls is movement variability caused by noise within the system. Both humans [23] and robots [24, 25] experience this.

In humans, stride to stride gait variability is correlated with fall rate [20, 26–28] and variability may be correlated with falling for robots as well [3]. Most studies have assumed that increased variability increases fall risk [3, 20, 26–28], although a few studies have suggested that the relationship is not that straightforward [29–32]. Prior studies have primarily examined the variability in step period. However, the variability in step period must arise from variation in leg movement, so this paper investigates joint angle variability.

Human gait variability (either for period or joint angles) has traditionally been modeled as random noise using standard deviations [33–36], while robot variability has largely been ignored. The variability is not entirely random, however. It is well established that there are fractal-like fluctuations in stride duration [27]. While joint angle variability may not contain this exact statistical structure [37], joint variability does contain some structure [38–40]. Further, joint angles are continuous, so joint variability cannot be treated as random selections from Gaussian distributions because those are discontinuous. Instead, joint angle variability can be modeled using a Fourier series which is continuous and can account for the structure in the variability [39]. Regardless of application, Fourier series are defined using a set of magnitude coefficients along with a fundamental frequency. Following the method in [39], each step can be modeled as having a different set of magnitude coefficients and fundamental frequency. It is then straightforward to describe the statistical properties of the variability using three parameter types—the standard deviation of the magnitude coefficients, the mean of the frequency, and the standard deviation of the frequency. (The mean of the magnitude coefficients is defined as zero.) All three parameter types could impact fall risk, although likely by differing amounts. Similarly, the variability at different joints may have different effects on fall risk.

To evaluate how the different variability aspects impact fall risk, a physics-based model that can fall (or otherwise fail) is needed. There are a wide variety of simulation approaches possible spanning a wide range of model and simulation complexity [41]. Since this work focuses on quantifying how different variability parameters affect fall risk, a moderately complex planar model that incorporates variability at each joint is appropriate. (This work uses a planar model is since most bipedal motion occurs in the sagittal plane.) The model used in this work is based on the bipedal robot control technique hybrid zero dynamics (HZD), which uses input-output linearization to track a commanded trajectory [42]. This modeling technique can produce gaits that predict healthy human walking [10], minimize robotic energy expenditure [24, 42], as well as handle discrete perturbations [43], model imperfections [25], and continuous imposed joint variability [11]. A key feature of HZD-based control is that is has at least one unactuated degree of freedom (DOF). Thus, the biped can fall forwards or backwards even when exactly tracking the commanded trajectory at the actuated joints.

This paper quantifies how different aspects of joint angle variability affect falling. To do so, the three parameters describing each joint’s variability were systematically altered within an HZD-based biped model simulation, and the number of steps to fall was analyzed to determine which factors had the most significant effect. Specifically, this paper tested the following hypotheses related to the number of steps to fall:

1. the magnitude of stance leg variability would have the most effect on fall risk because the stance leg is responsible for moving the center of mass and injecting energy into the next step;
2. the magnitude of swing leg variability would have a limited effect on fall risk because the swing leg is not in contact with the ground; and
3. the timing of the variability would have a limited effect on fall risk because it does not substantially alter the amount of energy injected into the step.

## Methods

### Model

**Fig 1:**
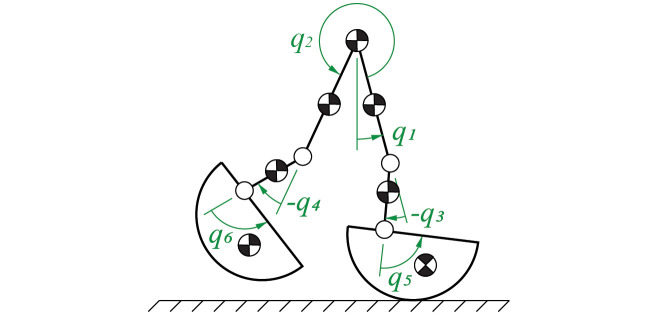
The planar six-link model used in this study. The stance hip angle *q*_1_ was unactuated. The remaining joints were actuated, where *q*_2_ was the swing hip angle, *q*_3_ was stance knee angle, *q*_4_ was the swing knee angle, *q*_5_ was the stance ankle angle, and *q*_6_ was the swing ankle angle.

An existing planar six link model was used and is briefly described here (Fig 1) [10, 11]. The six degree of freedom (DOF) model had a point mass at the hip. Each leg had knee and ankle joints; the two legs were connected via the hip joint. Thus, there were five actuated joints—stance (St) and swing (Sw) knee (K) and ankle (A) joints plus a swing hip (H) joint. Because this was a planar model, all joint angles represented flexion/extension and will simply be referred to as joint angles for conciseness. The five joints were actuated via ideal torque generators. The foot was modeled with a circular arc that rolls without slip, so the foot-ground interface was unactuated. Thus, the final DOF was the unactuated absolute angle of the biped. As a result, even when the actuated joints tracked the commanded motion perfectly, the biped could still fall because of the unactuated DOF’s movement.

Each step consisted of a finite-time, continuous stance period and an instantaneous, impulsive impact period during which the stance foot switched [24]. While humans have a finite time double support period, most HZD-based robots do not. Further, the relationship between double support and fall risk is unclear [4], so using the simpler instantaneous transition period did not automatically decrease either the model’s walking ability nor its stability.

The model used hybrid zero dynamics (HZD) to control the actuated joints’ movements [11, 42]. HZD-based control uses input-output linearization to track a commanded trajectory by encoding it in the output functions to be zeroed:

$$\begin{array}{c} y=h\left(q\right)=H_{0}q-h_{c}\left(s\left(q\right)\right) \end{array}$$ (1)

where *h* was a 5 dimensional vector-valued function to be zeroed, *H*_0_ was a matrix that mapped the joint angles to the actuated angles, *h*_*c*_ was a 5 dimensional vector-valued function of the commanded joint angles that included the variability, *q* was a vector of the 6 joint angles, and *s* was the phase variable. The phase variable defined the progression of the step; as the phase variable increased, the biped moved forward and the swing leg moved from behind the stance leg to in front of the stance leg. If the phase variable decreased, the biped moved backwards and the swing leg moved back behind the stance leg. For this work, the phase variable was chosen as the linearized hip position. The phase variable was normalized between 0 and 1 using:

$$\begin{array}{c} s\left(\theta \left(q\right)\right)=\frac{\theta -\theta_{P}^{+}}{\theta_{P}^{-}-\theta_{P}^{+}} \end{array}$$ (2)

where *θ* was the phase variable, $\theta_{P}^{+}$ was the phase variable at the start of the stance period, and $\theta_{P}^{-}$ was the phase variable at the end of the stance period [24]. When normalizing the phase variable, the nominal values for the periodic gait were used. The joint torques were found by performing input-output linearization on the equations of motion and output function using standard methods [44]. As a result, introducing variability into the output functions automatically introduced coupled variability into the joint torques.

**Fig 2:**
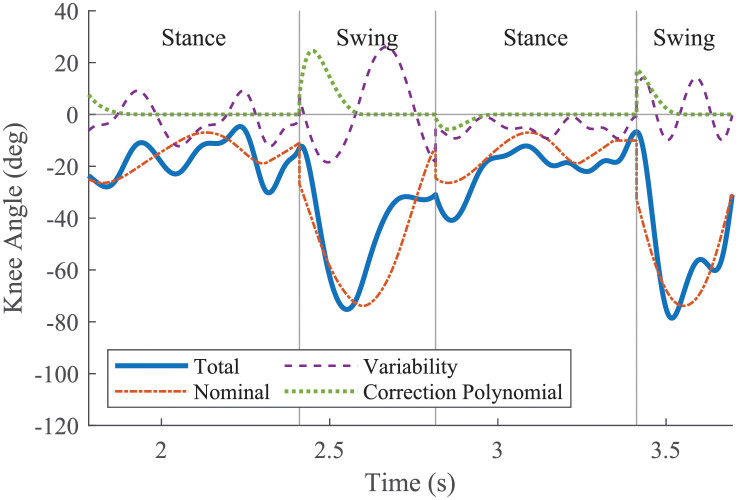
Total commanded motion. Total commanded motion of one knee for two consecutive strides with each component plotted separately. Negative angles indicate flexion. The nominal motion would be continuous if each step ended when planned. It repeats every stride. In contrast, the variability will typically be discontinuous between steps and is different for each step in the simulation. Because the total commanded motion must be strictly continuous, the correction polynomial was used to ensure continuity.

The commanded motion consisted of three components:

$$\begin{array}{c} h_{c}\left(s\left(q\right)\right)=h_{N}\left(s\right)+h_{V}\left(s\right)+h_{C}\left(s\right) \end{array}$$ (3)

where *h*_*c*_ was the total commanded joint angle, *h*_*N*_ defined the nominal periodic motion, *h*_*V*_ defined the joint variability, and *h*_*C*_ defined a correction polynomial to remove start of step error from the commanded motion and ensure the commanded motion was continuous (Fig 2) [11]. The gait would be periodic if *q*_*c*_ only consisted of *h*_*N*_. By varying *h*_*N*_, different gaits were achieved. By varying *h*_*V*_, different variability conditions were tested.

The nominal motion (*h*_*N*_) was defined using a fifth order Bézier polynomial with a constant velocity profile when *s* > 1 [11, 45]. Fifth order Bézier polynomials were used because they capture average human walking joint trajectories well in an HZD model [10]. While the nominal motion was defined such that the phase variable was normalized between *s* = 0 (start of stance period) and *s* = 1 (end of stance period), the addition of variability led to the phase variable obtaining values outside of this range. This, in turn, lead to the Bézier polynomial diverging to unwanted values when *s* > 1. The switch to a constant velocity profile resolved this issue. No special processing was performed when *s* < 0 since pilot testing indicated that the correction polynomial handled that well. Thus, the nominal motion was defined as:

$$\begin{array}{c} h_{N}\left(s\right)=\{\begin{array}{cc} \Sigma_{k=1}^{5}\alpha_{k}\frac{5!}{(5-k)!k!}s^{k}{\left(1-s\right)}^{5-k} & s\leq 1\\ \alpha_{5}+5\left(\alpha_{5}-\alpha_{4}\right)\left(s-1\right) & s>1 \end{array} \end{array}$$ (4)

where *α*_*k*_ were the polynomial coefficients chosen to minimize a weighted torque-square objective function [10]. Because previous research indicated that speed has an impact on falls [15, 21, 22, 46], three different nominal gaits from [10] were tested—slow (0.98 m/s), medium (1.30 m/s), and fast (1.56 m/s). The models associated with each gait were adult-sized, although each had somewhat different mass and length parameters because each gait was associated with a different person or group. The model for the slow speed had a mass of 73 kg and a leg length of 0.96 m. The model for the middle speed had a mass of 56 kg and a leg length of 0.90 m. The model for the fast speed had a mass of 52 kg and a leg length of 0.81 m. Other mass and length parameters were scaled using standard anthropometric tables [47, 48].

The variability was defined using Fourier series because it provides a convenient mathematical form for an arbitrary smooth function. Inspired by human joint variability [39], a 2^*nd*^-order Fourier series was used for the stance joints and a 1^*st*^-order Fourier series was used for the swing joints in this work:

$$\begin{array}{c} h_{V}\left(s\right)=a_{0}+\sum_{k=1}^{K}(a_{k}\;\text{cos}\left(k\omega s\right)+b_{k}\;\text{sin}\left(k\omega s\right)) \end{array}$$ (5)

where *a*_*k*_ and *b*_*k*_ were the magnitude coefficients, *ω* was the fundamental frequency, and *K* was the order of the series. Different coefficients and frequencies were chosen every step, resulting in a Gaussian distribution for each parameter. For each step, the frequency was chosen using a random draw. For the magnitude coefficients, the distributions had zero mean and specified standard deviations. Some magnitude coefficients were related using relationships based on human subject data [39]. Specifically, there was a linear relationship between the swing hip *a*_0_ and *b*_1_ coefficients, a linear relationship between the stance knee *b*_1_ and *b*_2_ coefficients, a linear relationship between the stance ankle *a*_0_, *b*_1_, and *a*_2_ coefficients, and a nonlinear relationship between the swing knee *a*_1_ and *b*_1_ coefficients. For coefficients with a relationship, one of the coefficients in the relationship was chosen using a random draw. The relationship was used to find nominal values of the other coefficients which were then perturbed using a random draw from a zero-mean Gaussian distribution with a standard deviation of 1°. For the remaining coefficients, the values were chosen using random draws. While this variability format was inspired by human motion [39], and has been shown to well represent it, Fourier series are very general, so the results likely apply to robotic systems as well.

Because of the variability, there were usually differences between the commanded and actual joint states at the start of a step. A fifth-order polynomial removed them and then smoothly decayed to 0 for *s* ≥ 0.5 [49].

### Analysis procedures

To quantify how variability affected fall risk, extensive simulations were performed while systematically varying the variability parameters.

#### Factors

Only the Fourier series representing the variability (Eq 5) were explicitly altered in this study. Specifically, the magnitude and frequency coefficient distributions were altered to analyze the effect of each aspect of variability on falling. The variability magnitude described the amount the joint angle deviated from the nominal angle. The variability frequency described how quickly the commanded angle oscillated around the nominal joint trajectory. This is related to the timing of the variability, which has been shown to change throughout the gait cycle [50]. In practice, altering the variability frequency largely seemed to alter the joint velocity. The following factors were altered for each actuated joint for a total of fifteen factors.

- Magnitude (Mag): the standard deviation of the Gaussian distribution for the magnitude coefficients describing the variability. The mean is zero and therefore does not need to be altered.
- Frequency Mean (FMean): mean of the Gaussian distribution for the fundamental frequency describing the variability.
- Frequency Standard Deviation (FStd): standard deviation of the Gaussian distribution for the fundamental frequency describing the variability.

All of these factors only alter the variability. Humans walk millions of steps before falling, so to make the study computationally feasible, factor values were chosen so that the model fell more quickly. To determine the values, feasibility simulations were performed by altering the values of each joint individually. The goal was to incorporate enough variability so that the model fell relatively quickly, while ensuring that the model still took some steps (> 20). Two levels for each factor were chosen, low in which the biped took many steps (> 50) and high in which the biped took a few steps (< 50) (Table 1). The standard deviations for *a*_2_ and *b*_2_ were half of the standard deviation of the other coefficients. This generally results in smaller values for *a*_2_ and *b*_2_ as is typical in Fourier series and as is seen for human joint variability [39]. Compared to human values, the chosen values were up to eight times larger for magnitude, three times larger for frequency mean, and five times larger for frequency standard deviation. To ensure mostly positive frequency values, frequency standard deviation was set to 1/3 of frequency mean.

**Table 1:** Variability parameter values. Magnitude, frequency mean, and frequency standard deviations values used in the experiment at both the low and high levels. For the magnitude coefficients, the second order coefficients *a*_2_ and *b*_2_ were half the value given in the table. The low and high levels for the magnitude of the stance ankle variability were different from the rest of the joints. *Key: St = stance, Sw = swing, H = hip, K = knee, A = ankle*.

Variable	Joint	Low Level	High Level
Magnitude	StK, SwH, SwK, SwA	4°	8°
	StA	0.5°	2°
Frequency Mean	StK, StA, SwH, SwK, SwA	3°/% step	15°/% step
Frequency Standard Deviation	StK, StA, SwH, SwK, SwA	1°/% step	5°/% step

#### Design of experiment

Because of the number of factors, it was not feasible to test every possible combination, so a fractional factorial design was used [51]. Fractional factorial designs intelligently choose factor combinations to test, reducing the total number of experiments. This allows the analysis of main effects (when a factor by itself has a significant influence on the outcome) as well as interactions (when multiple factors together have a significant influence). As is typical, many higher order interactions were assumed negligible, allowing a fractional factorial design. Because a fractional design does not test all combinations, some effects were aliased, where the measured effect includes the influence of one or more actual effects and cannot be estimated separately from each other. A resolution IV design was used, meaning that at most main effects were aliased with three-factor interactions.

To determine steps to fall, simulations were performed until the biped fell or took 500 steps. For each condition, the simulation was repeated 10 times to account for the stochastic nature of the system. The median number of steps before falling was used in the analysis.

#### Evaluation methods

To evaluate the hypotheses, Pareto charts and an ANOVA analysis (Minitab, State College, PA) were used to determine what factors and interactions had a significant effect on falling (*α* = 0.05). The median number of steps for each run was used to determine the significant factors. Pareto charts for each speed were generated; speed was not included as a factor. Pareto charts visually depict the statistically significant factors. To do so, they use t-statistics to compare the absolute standardized effects against the null hypothesis that the effect is 0, so a larger t-statistic for a factor indicates a more significant effect on falling. Factors were ordered from largest to smallest t-statistic and plotted. A t-statistic reference line indicating the border of significance was included; any factors that crossed the line indicate statistical significance.

For significant factors, additional analysis was performed to further quantify its impact. Specifically, the average number of steps was calculated when the factor was at the low (or high) value across all runs (set of 10 simulations all with the same speed and factor values) allowing for a linear fit. This parameter will be referred to as “slope” throughout the rest of the paper. The slope’s absolute value indicated how changing the factor changed steps to fall; the greater the slope, the greater the change in steps to fall. The slope’s sign indicated whether the factor had a positive effect (the number of steps increased as the factor increased) or a negative effect (the number of steps decreased as the factor increased). Since increased variability is generally assumed to decrease the number of steps to fall (i.e., make the biped more unstable), the slopes were generally expected to be negative.

An ANOVA was used to confirm the results of the Pareto chart as well as incorporate speed as an additional factor. Within the ANOVA analysis, only main effects, speed, and interactions between speed and other main effects were analyzed. Factors and interactions that had a p-value ≤ 0.05 were statistically significant.

## Results

In general, steps to fall increased as speed increased. The model generally took 2–20 steps for the slow speed, 5–40 steps for the middle speed, and 10–50+ steps for the fastest speed. The slow and middle speeds never completed 500 steps (maximum possible) while the fast speed completed 500 steps 49 times.

**Fig 3:**
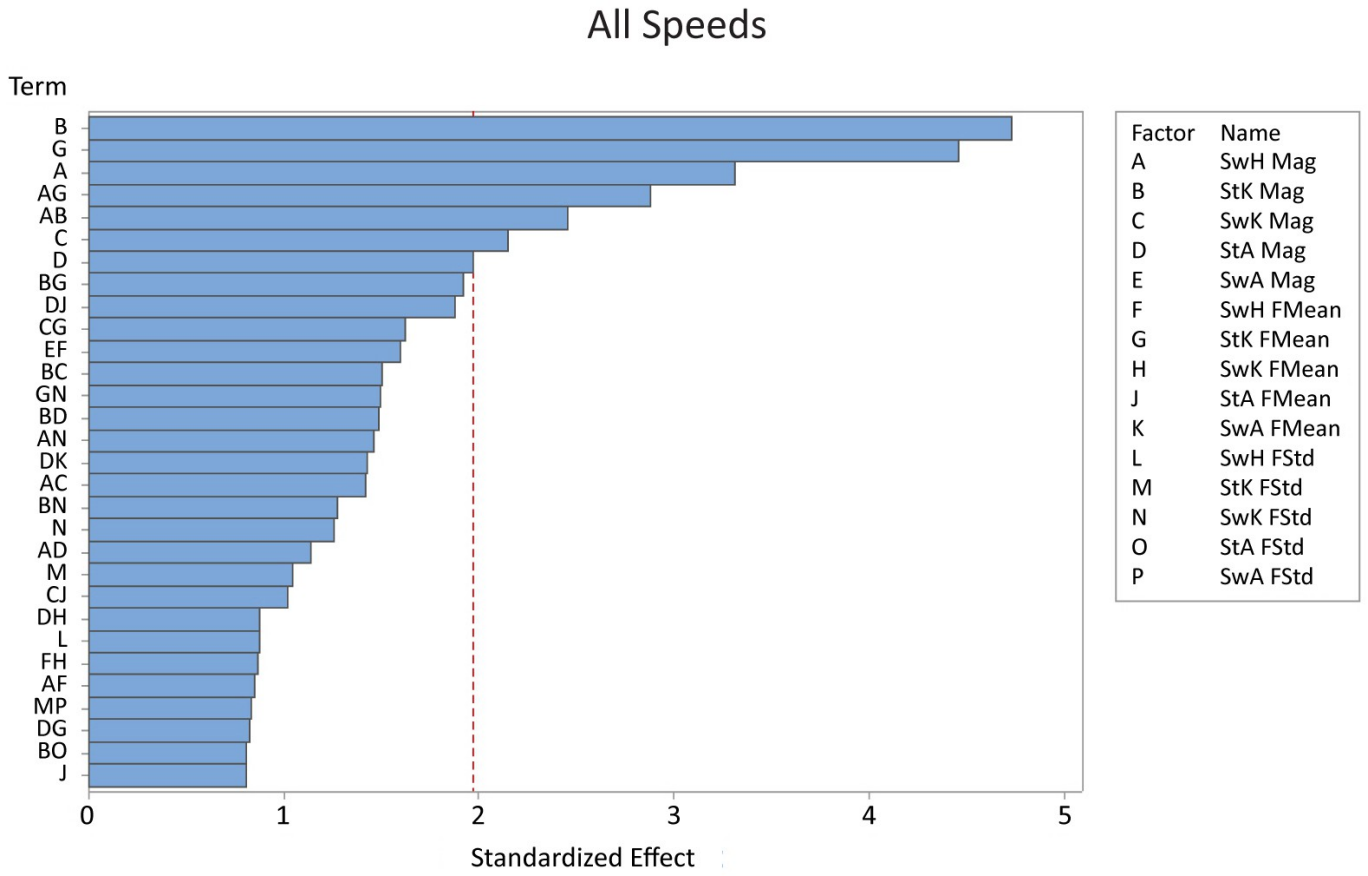
The Pareto chart with the 30 most significant factors on the number of steps before a fall across all three speeds. Out of a total of seven significant factors, stance knee magnitude and stance knee frequency mean were the most significant factors when all three speeds were analyzed together. *Key: St = stance, Sw = swing, H = hip, K = knee, A = ankle, Mag = magnitude, FMean = frequency mean, FStd = frequency standard deviation*.

For all speeds combined, stance knee magnitude, stance knee frequency mean, and swing hip magnitude had the largest effect on falling (Fig 3, Table 2). All significant factors decreased steps to fall as the variability increased. Stance knee magnitude had the most significant effect on falling; on average, the model took 31 more steps when at the low level of variability compared to the high level of variability when combining results from all speeds. The stance knee frequency mean had almost as large of an effect, with the model taking 29 more steps on average when at the low level of variability compared to the high level of variability.

**Table 2:** The slope for all significant factors on the number of steps before a fall across all three speeds. Interactions are indicated with a “+”. The slope indicates the magnitude and sign of the effect, where a negative slope indicates that the number of steps taken decreases as the variability changes from low to high. As expected, all slopes were negative.

Factor	Slope
Stance Knee Magnitude	-15.4
Stance Knee Frequency Mean	-14.5
Swing Hip Magnitude	-10.8
Swing Hip Magnitude + Stance Knee Frequency Mean	-9.4
Swing Hip Magnitude + Stance Knee Magnitude	-8.0
Swing Knee Magnitude	-7.0
Stance Ankle Magnitude	-6.4

**Fig 4:**
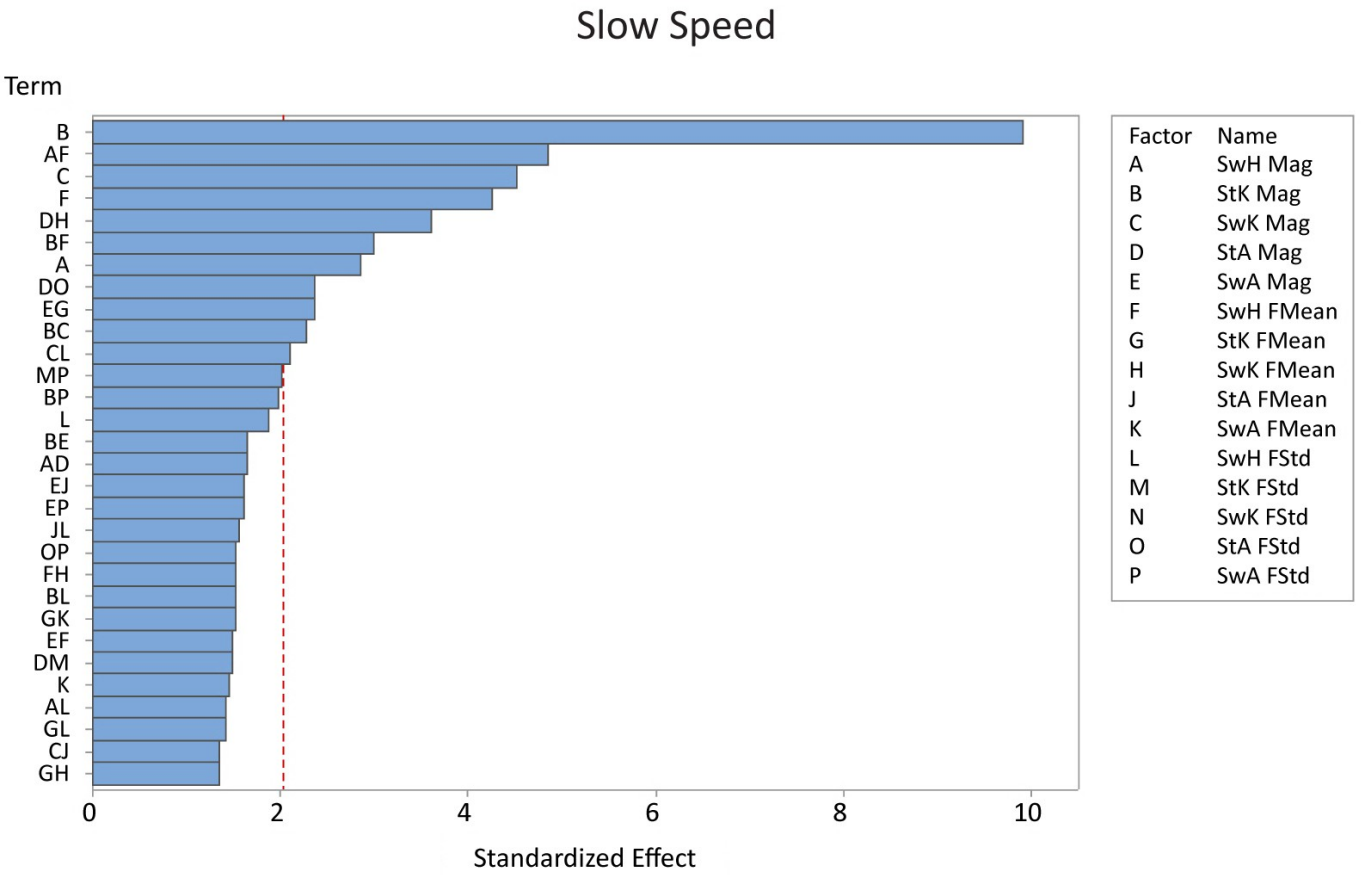
The Pareto chart with the 30 most significant factors on the number of steps before a fall for the slow speed. By far the most significant factor was stance knee magnitude, although there were a total of eleven significant factors. *Key: St = stance, Sw = swing, H = hip, K = knee, A = ankle, Mag = magnitude, FMean = frequency mean, FStd = frequency standard deviation*.

For the slow speed, stance knee magnitude, the interaction between swing hip magnitude & swing hip frequency mean, and swing knee magnitude had the largest effect on falling (Fig 4). Stance knee magnitude was approximately twice as significant as the next two factors, indicating that varying this factor changed steps to fall the most. Increasing stance knee magnitude from the low to the high value decreased the number of steps by 8 steps on average, a change of approximately 60% (Table 3). While increasing stance knee magnitude variability decreased the number of steps taken, over half of the factors increased steps to fall as variability increased for the slow speed.

**Table 3:** The slope for all significant factors for the slow speed. Interactions are indicated with a “+”. While the slopes were expected to be negative (indicating that the model fell more quickly at high levels of variability), approximately half of the slopes were positive.

Factor	Slope
Stance Knee Magnitude	-4.1
Swing Hip Magnitude + Swing Hip Frequency Mean	2
Swing Knee Magnitude	1.9
Swing Hip Frequency Mean	1.8
Stance Ankle Magnitude + Swing Knee Frequency Mean	-1.5
Stance Knee Magnitude + Swing Hip Frequency Mean	-1.2
Swing Hip Magnitude	1.2
Stance Ankle Magnitude + Stance Ankle Frequency Standard Deviation	-1
Swing Ankle Magnitude + Swing Knee Magnitude	1
Stance Knee Magnitude + Swing Knee Magnitude	-0.9
Swing Knee Magnitude + Swing Hip Frequency Standard Deviation	0.9

**Fig 5:**
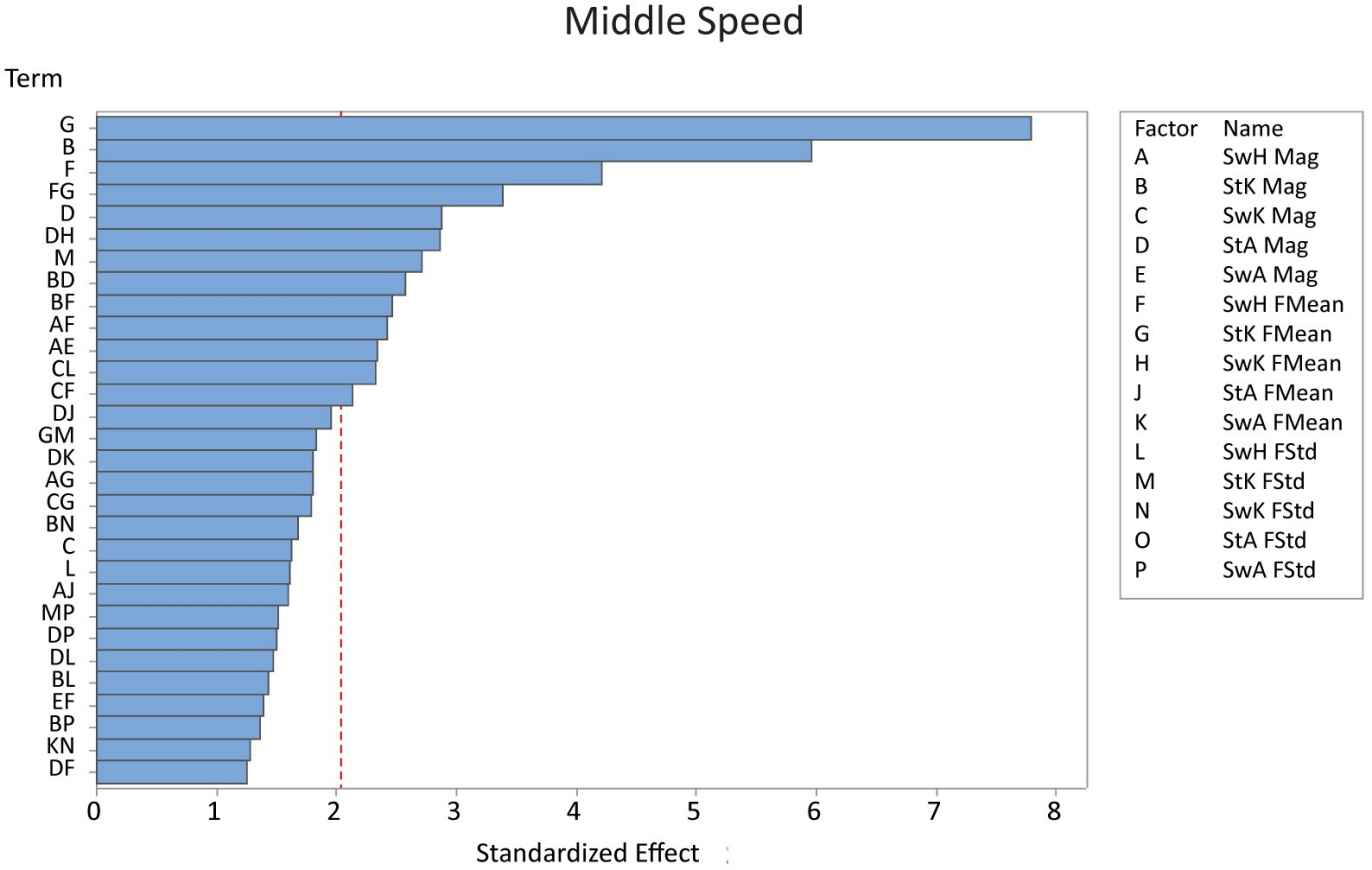
The Pareto chart showing the 30 most significant factors on the number of steps before a fall for the middle speed. Stance knee frequency mean and stance knee magnitude were the most significant factors out of thirteen total significant factors. *Key: St = stance, Sw = swing, H = hip, K = knee, A = ankle, Mag = magnitude, FMean = frequency mean, FStd = frequency standard deviation*.

For the middle speed, stance knee frequency mean, stance knee magnitude, and swing hip frequency mean had the largest effects on falling (Fig 5). Varying the stance knee frequency mean resulted in the model taking 15 more steps on average when at the low level of variability, while decreasing the stance knee magnitude variability and swing hip frequency mean resulted in an additional 11 and 8 steps on average before falling, respectively (Table 4). As the variability increased for the middle speed, all significant main effects decreased steps to fall, while most significant interactions increased steps to fall by 25–40%.

**Table 4:** The slope for all significant factors for the medium speed. Interactions are indicated with a “+”. When changing just one factor, the slopes are negative, indicating that the model falls more quickly when that aspect of variability is increased.

Factor	Slope
Stance Knee Frequency Mean	-7.4
Stance Knee Magnitude	-5.6
Swing Hip Frequency Mean	-4
Swing Hip Frequency Mean + Stance Knee Frequency Mean	3.2
Stance Ankle Magnitude	-2.7
Stance Ankle Magnitude + Swing Knee Frequency Mean	-2.7
Stance Knee Frequency Standard Deviation	-2.6
Stance Knee Magnitude + Stance Ankle Magnitude	2.4
Stance Knee Magnitude + Swing Hip Frequency Mean	2.3
Swing Hip Frequency Mean + Swing Hip Frequency Mean	2.3
Swing Hip Magnitude + Swing Ankle Magnitude	-2.2
Swing Knee Magnitude + Swing Hip Frequency Standard Deviation	2.2
Swing Knee Magnitude + Swing Hip Frequency Mean	2

**Fig 6:**
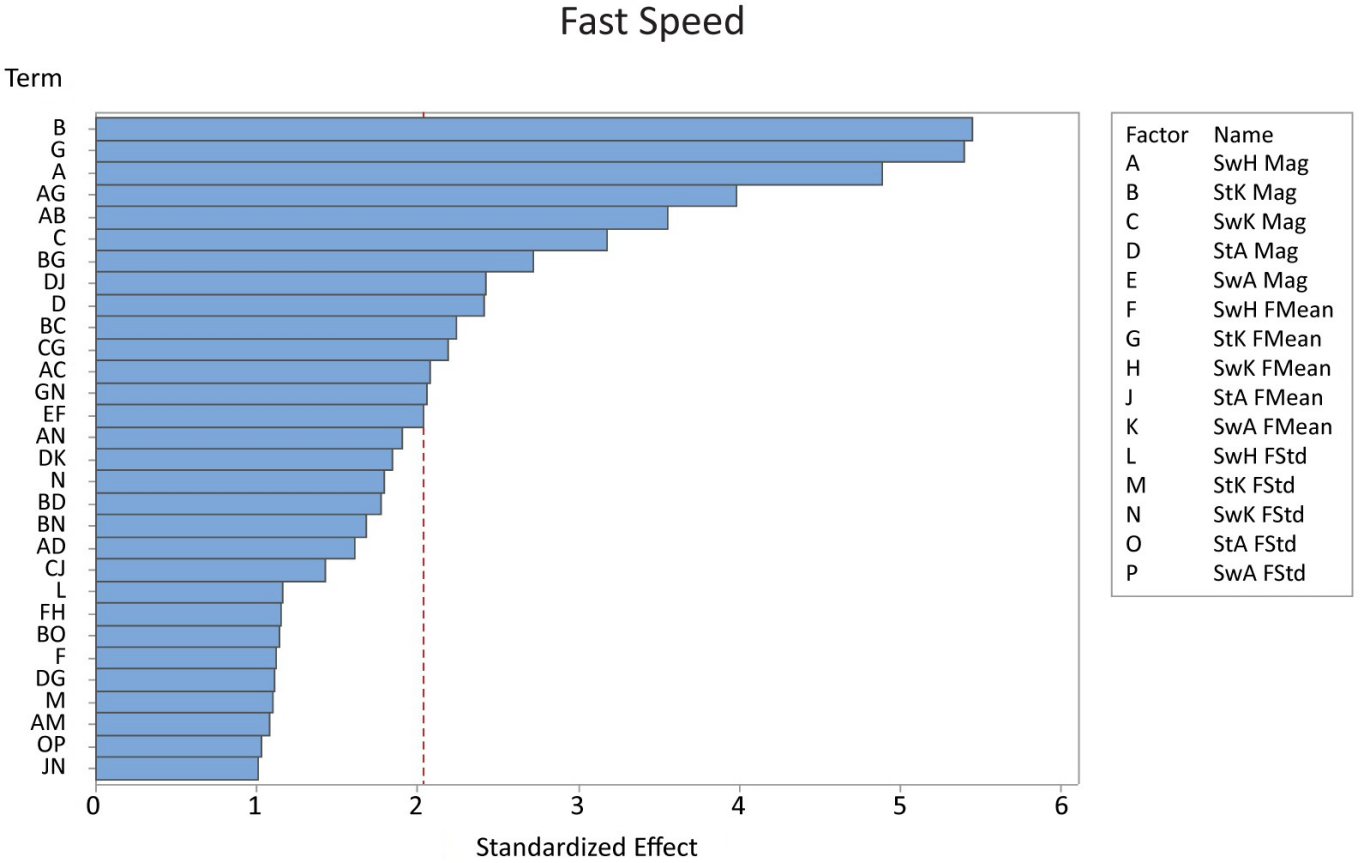
The Pareto chart showing the 30 most significant factors on the number of steps before a fall for the fastest speed. Out of a total of fourteen significant factors, stance knee magnitude, stance knee frequency mean, and swing hip magnitude were the most significant. *Key: St = stance, Sw = swing, H = hip, K = knee, A = ankle, Mag = magnitude, FMean = frequency mean, FStd = frequency standard deviation*.

For the fast speed, stance knee magnitude, stance knee frequency mean, and swing hip magnitude had the largest effects on falling (Fig 6). Stance knee magnitude and stance knee frequency mean had about the same effect on falling; changing stance knee magnitude resulted in a difference of 73 steps to fall on average between low and high variability while changing the stance knee frequency mean resulted in a difference of 72 steps to fall on average (Table 5). Changing the swing hip magnitude resulted in a difference of 65 steps to fall on average. Thus, increasing the top three factors all decreased steps to fall by approximately 70% for the fast speed. Similar to the middle speed, all significant main effects decreased steps to fall, while most significant interactions increased steps to fall as variability increased.

**Table 5:** The slope for all significant factors for the fast speed. Interactions are indicated with a “+”. The slopes for the main effects (when one factor was varied) tended to be negative as expected. In contrast, many of the interactions were positive, indicating that the combination of factors did not reduce number of steps to fall as variability increased as much as would be expected from just the main effects.

Factor	Slope
Stance Knee Magnitude	-36.5
Stance Knee Frequency Mean	-36.2
Swing Hip Magnitude	-32.8
Swing Hip Magnitude + Stance Knee Frequency Mean	26.7
Swing Hip Magnitude + Stance Knee Magnitude	23.8
Swing Knee Magnitude	-21.3
Stance Knee Magnitude + Stance Knee Frequency Mean	18.1
Stance Ankle Magnitude + Stance Ankle Frequency Mean	-16.3
Stance Ankle Magnitude	-16.2
Stance Knee Magnitude + Swing Knee Magnitude	15
Swing Knee Magnitude + Stance Knee Frequency Mean	14.7
Swing Hip Magnitude + Swing Knee Magnitude	13.9
Stance Knee Frequency Mean + Swing Knee Frequency Standard Deviation	12.9
Swing Ankle Magnitude + Swing Hip Frequency Mean	5.6

To verify that speed interacted significantly with the variability factors, an ANOVA was completed. Speed, the magnitude for all joints except the swing ankle, and stance knee frequency mean were significant factors (Table 6). Additionally, the interaction between speed and the above factors were significant. Most significant factors in the ANOVA were also significant in the Pareto charts (Table 7).

**Table 6:** ANOVA analysis including speed as a factor. For conciseness, all factors and interactions that had a p-value > 0.1 were omitted from the table. The significant factors in the ANOVA analysis included the magnitude of all joints except for the swing ankle, the frequency mean for the stance knee, the speed, and the interaction between speed and all factors mentioned above.

Source	p-Value
Swing hip magnitude	≪ 0.001
Stance knee magnitude	≪ 0.001
Swing knee magnitude	0.006
Stance ankle magnitude	0.011
Stance knee frequency mean	≪ 0.001
Speed	≪ 0.001
Swing hip magnitude × Speed	≪ 0.001
Stance knee magnitude × Speed	≪ 0.001
Swing knee magnitude × Speed	≪ 0.001
Stance ankle magnitude × Speed	0.022
Stance knee frequency mean × Speed	≪ 0.001
Swing knee frequency standard deviation × Speed	0.085

**Table 7:** All significant factors across the different analyses. Factors that are significant in at least three analyses are indicated with *. The main effects of the magnitude factors were found across most of the analyses, while frequency factors and interactions were more likely to only be found in one of the analyses. *Key: St = stance, Sw = swing, H = hip, K = knee, A = ankle, Mag = magnitude, FMean = frequency mean, FStd = frequency standard deviation*.

Factor	Pareto				ANOVA
	All Speeds	Slow	Middle	Fast	
SwH Mag *	Yes	Yes	No	Yes	Yes
StK Mag *	Yes	Yes	Yes	Yes	Yes
SwK Mag *	Yes	Yes	No	Yes	Yes
StA Mag *	Yes	No	Yes	Yes	Yes
SwH FMean	No	Yes	Yes	No	No
StK FMean *	Yes	No	Yes	Yes	Yes
StK FStd	No	No	Yes	No	No
SwH Mag + StK Mag	Yes	No	No	Yes	-
SwH Mag + SwK Mag	No	No	No	Yes	-
SwH Mag + SwA Mag	No	No	Yes	No	-
SwH Mag + SwH FMean	No	Yes	Yes	No	-
SwH Mag + StK FMean	Yes	No	No	Yes	-
StK Mag + SwK Mag	No	Yes	No	Yes	-
StK Mag + StA Mag	No	No	Yes	No	-
StK Mag + SwH FMean	No	Yes	Yes	No	-
StK Mag + StK FMean	No	No	No	Yes	-
SwK Mag + SwH FMean	No	No	Yes	No	-
SwK Mag + StK FMean	No	No	No	Yes	-
SwK Mag + SwH FStd	No	Yes	Yes	No	-
StA Mag + SwK FMean	No	Yes	Yes	No	-
StA Mag + StA FMean	No	No	No	Yes	-
StA Mag + StA FStd	No	Yes	No	No	-
SwA Mag + SwH FMean	No	No	No	Yes	-
SwA Mag + StK FMean	No	Yes	No	No	-
SwH FMean + StK FMean	No	No	Yes	No	-
StK FMean + SwK FStd	No	No	No	Yes	-

## Discussion

Factors that significantly affected falling were somewhat different for each speed (Table 7). In most cases, only main effects were significant at more than one speed. Depending on the condition, going from the low to high value of variability changed the number of steps by 10–70%. In general, steps to fall decreased as variability increased for the main effects, indicating that increasing variability increased fall risk. This is consistent with previous work showing that increased variability is correlated with increased fall risk [3, 20, 26–28]. However, in a few cases, increasing variability decreased fall risk. This may also be consistent with previous work showing there is an optimal level of variability and values either above or below that level increased fall risk [29]. These results are somewhat in contrast to prior work in which normally-distributed noise was applied to the swing hip of the simplest walking model [32]. In that study, increasing noise increased variability in step length and period but had little effect on stability. While few of the interactions in this study were significant, approximately half of the significant interactions increased steps to fall as variability increased, indicating that a combination of increased variability may not affect the steps to fall as significantly as the main effects themselves would predict. In other words, this may mean that the effect of variability at one joint can be partially canceled out by variability at a different joint.

Except for the swing ankle, the magnitude factors consistently had the most significant effect on steps to fall for the values tested (Table 7); most were significant for two of the three speeds. Further, these factors were a part of many significant interactions. This suggests that altering variability magnitude has the most significant effect on falling and confirms our first hypothesis. Stance knee magnitude was the most significant factor and decreased steps to fall by 50–70% as the variability increased. In other words, doubling the variability approximately doubled the fall risk. It was the only factor that was significant in every analysis (Table 7) and was a top factor in every Pareto chart. The stance knee appears to position the back leg so that both the angle between the point of contact and the hip and the hip’s velocity vector are aligned appropriately for a good step-to-step transition. Increased knee extension, and sometimes hyperextension, caused the biped to stall and fall backward as there was insufficient momentum to progress over the extended stance knee. Hyperextension was most likely at the slowest speed because the mean angle was closer to zero, so a smaller change was required. Increased stance knee flexion often resulted in a longer step and increased extension of the next step. This suggests that reducing knee variability in the absence of other control actions could reduce backwards falls.

The relative importance of stance ankle variability on steps to fall was inconclusive. For the values used in the full study, stance ankle variability magnitude did not impact steps to fall as much as the stance knee variability magnitude did. For all three speeds, increasing stance ankle magnitude decreased the number of steps to fall by 25–40%, but was not a top factor for any Pareto chart. The apparent lack of importance could partly be because the absolute change in ankle variability magnitude was smaller than for the other joints, although the relative change was larger. This is in contrast to the results from the feasibility study which indicated that stance ankle magnitude values had a much larger effect and therefore required lower magnitude values than the for other joints (Table 1). Previous studies have also found that stance ankle motion significantly contributes to start-of-step energy [11, 52–54], possibly due to the proximity of the stance ankle to ground. The inconsistency between the feasibility study results and the full study results suggests a nonlinear relationship between stance ankle magnitude and steps to fall. This is perhaps not surprising, given that the simplest walking model appears to have a nonlinear relationship between noise at the joint level and variability in step length and period [32]. In terms of shape of the nonlinearity, one possibility is that stance ankle magnitude is moderately important for low values and suddenly becomes very important at higher values. The other possibility is that the impact of variability magnitude grows smoothly. Unfortunately, only two values of stance ankle magnitude were used in this study, so it was only possible to find a linear relationship. Further work is required to fully determine the effect of stance ankle magnitude on falling, but these results suggest that stance ankle magnitude has a moderate effect on steps to fall at low values of stance ankle magnitude variability and a large effect at higher values of stance ankle magnitude variability.

Swing hip magnitude primarily affected swing leg timing and changed steps to fall by 20–70%. For all speeds combined and the fast speed, increasing swing hip magnitude variability decreased steps to fall by 50–70% (i.e., it increased fall risk). In contrast, steps to fall increased by 30% as variability increased for the slow speed (i.e., it decreased fall risk). With higher variability, the biped often fell backward as the swing leg mass was too far behind the point of contact. Occasionally, the biped fell forward, as the increased hip variability increased the swing leg acceleration and caused the biped to trip.

Swing knee and ankle magnitude were not originally expected to impact falling because the swing leg does not contact the ground. However, swing knee magnitude was a significant factor possibly because knee variability just before foot contact affected the stance knee’s next step. In other words, the variability during swing may not matter, but the altered position at heel contact may. Another possibility is that the swing knee’s variability altered the biped’s overall momentum, causing it to speed up or slow down, and therefore making it easier or harder to complete the step. Increasing swing knee magnitude decreased steps to fall by 40–50% for all speeds combined and the fast speed, but increased steps to fall by 50% for the slow speed. In contrast, the swing ankle was not a significant factor. Because of the split results, our second hypothesis cannot be confirmed or rejected.

Our results support the third hypothesis that variability frequency has little effect on steps to fall. While the frequency mean, particularly for the stance knee, had some significant results, no frequency mean component was significant for all speeds, indicating that the variability frequency has less of an impact than the variability magnitude. It appears that the main impact of increasing the frequency mean is to increase the velocity difference between the periodic and variable gait. The frequency standard deviation had little effect on falling. Because it increases the number of steps with both higher and lower frequencies, this is not surprising, particularly since the variability frequency in general does not have much of an effect. This is consistent with human subject studies that found that fall risk was not correlated with variation in the timing of muscle activations [55]. Thus, our third hypothesis is confirmed as the timing of the variability (frequency) did not have as large of an impact as the variability magnitude.

In addition to the variability components, speed also had a significant effect on steps to fall. Previous studies have indicated that slower walking may increase [56, 57] or decrease stability [15, 21, 22, 46]. For this study, the biped tended to fall more quickly at slower speeds because it had less momentum in general, so variability was more likely to result in insufficient momentum and a backwards fall.

While certain aspects of the model were based on human gait, the results do not, and were not expected to, directly correlate with falls in humans. The model fell much more frequently than humans do by design. We chose variability parameters, particularly for the variability magnitudes, that were much larger than observed in human walking. Given the apparent nonlinearity in the relationship between ankle variability and fall risk, a future study should investigate how fall risk changes as variability magnitude increases from very low values to very high values for all joints. Further, when the variability caused the phase variable to drop below zero or rise above one, no effort was made to ensure the nominal motion remained humanlike. As a result, the variability could have an exaggerated effect at the beginning and end of some steps if both the nominal and variability terms in the commanded motion diverged from periodic walking motions. In addition, unlike the model, humans actively redistribute forces to compensate for undesirable joint positions. Studies have observed intra-joint coordination between the hip and the knee [58] as well as between the knee and the ankle [38]. Similarly, humans react to falling using their whole body [59] while the model’s controller had no explicit fall prevention action. Further, humans appear to modulate their variability for a variety of tasks so that task-relevant variability is much smaller than task-irrelevant variability [60, 61]. Thus, the active control in humans likely allows them to adapt and prevent many of the falls that the model experienced. Instead, this study provides insight into which aspects of variability are most likely to cause falls in the absence of active control to reject or modulate the variability.

Overall, this simulation study found that, except at the swing ankle, the variability magnitude had the most significant effect on steps to fall for the values tested. Stance knee variability magnitude was the most significant factor for the values tested. In addition, speed was a significant factor. This suggests that increasing the joint variability magnitude may increase fall risk, particularly if the controller is not able to actively compensate. Because the variability frequency generally does not affect fall risk, future studies should focus on further exploring the likely nonlinear relationship between variability magnitude and fall risk.

## Supporting information

S1 File Code used to generate simulations. Matlab code used to generate the simulations.
(ZIP)
