# Supplementary material for: Quantifying the effect of sagittal plane joint angle variability on bipedal fall risk
Source: PLoS One. 2022 Jan 26;17(1):e0262749. doi: 10.1371/journal.pone.0262749 (PMC8791504; doi:10.1371/journal.pone.0262749)

Presentation Format Function

Overview

Function to prepare a figure for a presentation or paper. This function can increases the width of the lines, change the color of lines, change the font type, change the font size, change the marker size, change the marker coloring, update labels and legends, resize the figure and save it. This file can save as a tex/eps file combination (for Latex), as an .emf file (for Microsoft Office) or as a .fig file. Both eps and emf are vector graphic formats, so the file can be saved at the size needed for the document without worry about the resolution of the figure. This makes it easier to ensure all figure text is readable in the document.

```
presentationFormat(figHandle, lineHandles, savefile, plotSize, userOptions)
```

Inputs:

- figHandle: the handle to the figure. To get the handle of the current figure, use `gcf`
- lineHandles: handles all of the lineseries objects in the figure. These can be obtained using `findall(gcf, 'type', 'line')`
- savefile: the name of the file to save the figure to or the path plus filename.
- plotSize: a row vector containing the desired width and height of the figure in cm. For a single column figure, 9x6 cm works well. For a figure spanning the width of the page, 18x8 cm works well. To leave the figure at the current size, pass in an empty vector `[]`.
- userOptions: a data structure containing information about the desired final plot. All fields are optional.
    - sets: use to group lines together. A vector containing the length of each set. In `lineHandles`, each set of data must be grouped together. The number of sets is N. A further discussion can be found below.
    - fontSize: the size of the font, a scalar. Default is 10.
    - font: the font. Does not affect the Latex version of the saved figure. Default is current font.
    - xlabel: the text for the xlabel. If included, this replaces the current xlabel.
    - latexXlabel: The xlabel for the Latex version of the saved figure. This is particularly useful if the xlabel contains mathematical symbols.
    - ylabel: the text for the ylabel. If included, this replaces the current ylabel.
    - latexYlabel: The ylabel for the Latex version of the saved figure. This is particularly useful if the ylabel contains mathematical symbols.
    - title: the text for the title. If included, this replaces the current title.
    - latexTitle: The title for the Latex version of the saved figure. This is particularly useful if the title contains mathematical symbols.
    - legend: either the position of the legend, true to place a legend in the default position or false for no legend.
    - legendEntries: the text for the legend entries. If included, this replaces the current legend entries.

- latexLegendEntries: The legend entries for the Latex version of the saved figure. This is particularly useful if the legend contains mathematical symbols.
- lineWidth: the width of the lines, default is 1.25. This can either be a scalar value which will change the width of all lines, or a vector with a legnth of n or N.
- markerSize: the size of the markers, default is 6. This can either be a scalar value which will change the size of all markers, or a vector with a legnth of n or N.
- color: a matrix of colors for the lines. This can either be a 1x3 row vector, a nx3 matrix or an Nx3 matrix. To get easily distinguished colors, use the function `distinguishable_colors()` (http://www.mathworks.com/matlabcentral/fileexchange/29702-generate-maximally-perceptually-distinct-colors).
- markerEdgeColor: a matrix of colors for the marker edges. This can either be a 1x3 row vector, a nx3 matrix or an Nx3 matrix.
- markerFaceColor: a matrix of colors for the marker faces. This can either be a 1x3 row vector, a nx3 matrix or an Nx3 matrix.
- keepLineWidth: If true, don't change the width of the lines. Default is false.
- keepMarkerSize: If true, don't change the marker size. Default is false.
- keepFontSize: If true, don't change the font size. Default is false.
- saveLatex: If true, save a set of .tex/.eps files for a latex document using `matlabfrag` (http://www.mathworks.com/matlabcentral/fileexchange/21286-matlabfrag). Default is true.
- saveMatlab: If true, save a fig file. Default is true.
- saveEMF: If true, save an EMF file. Default is false.

Figures and Latex

There are several packages for Latex that allows the text for a graphic to be compiled using the pdfLatex compiler. This has several advantages, including the ability to use all of Latex functionality, such as mathematical typesetting and automatic labeling, within graphics. In addition, it results in uniform font between the document text and the graphics. In order for this to work, the graphic's text is stored in a different file than the graphic's image. There are several functions on the Matlab file exchange which automatically separate plot text from the plot itself. The function `presentationFormat` uses is `matlabfrag` (http://www.mathworks.com/matlabcentral/fileexchange/21286-matlabfrag). To put the files back together in Latex, use the package pstool (http://www.ctan.org/pkg/pstool). The following gives a minimal example:

```
\documentclass{article}
\usepackage{pstool}  % Load the package
\begin{document}
\psfragfig{myfig}  % Put the graphic back together
\resizebox{width}{height}{\psfragfig{myfig}}  % Put the graphic back together and adjusts
    the sizing. To keep the aspect ratio, specify either width or height, and set the other value as !.
\end{document}
```

The document must be compiled with pdfLatex and the `-shell-escape` option. Once the graphic has been compiled, a pdf version of the graphic with the text and image combined is created.

The Sets Functionality

In general, the userOptions fields can be broken into two types, those that apply to the whole figure and those that can be different for different lines. For example, the field *fontSize* applies to the whole figure while the field *color* can specify different colors for each line handle. For the second type of field, either a single value is given for all of the line handles or a vector or matrix with a height equal to the number of lines or sets is given. Sets can are groups of line handles that should all share the same properties. The figure below shows two sets of discontinuous data (blue and magenta). To plot without vertical lines, each continuous segment has its own line handle, which are 1 through 5 in this example. However, it is much more intuitive to treat lines 1, 2 and 3 as a single line and lines 4 and 5 as a second line. Each grouping of lines, for example 1, 2 and 3, is referred to as a set. Sets are defined using the userOptions field called *sets*, which is a vector containing the length of each set. The sum of the field *sets* must equal the number of line handles. So, for the figure below, the value of *sets* would be [3 2]. For the grouping to work properly, the lineHandles vector must contain the handles for lines 1, 2 & 3 and then the handles for lines 4 & 5.

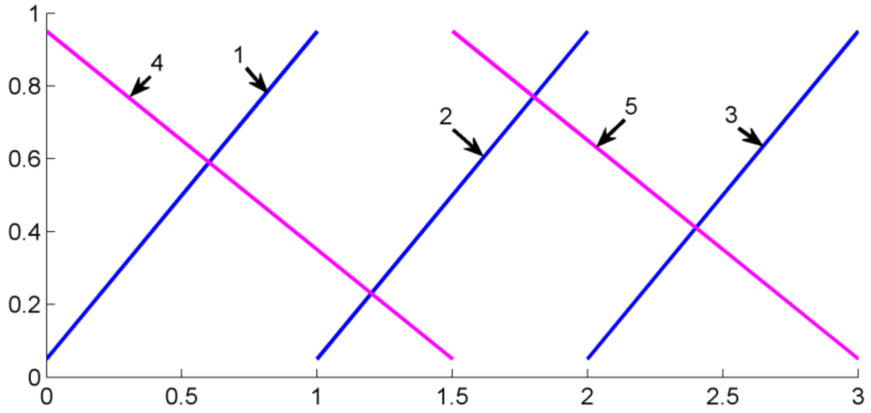

Supplement: S1 File — Matlab code used to generate the simulations. (ZIP) [file pone.0262749.s001.zip › S1_File/RADIUS/models/HEALTHY_HUMAN_REAL_ANKLE/docs/Presentation Format Users Guide.pdf]
