# Supplementary material for: Quantifying the effect of sagittal plane joint angle variability on bipedal fall risk
Source: PLoS One. 2022 Jan 26;17(1):e0262749. doi: 10.1371/journal.pone.0262749 (PMC8791504; doi:10.1371/journal.pone.0262749)
Supplement: S1 File — Matlab code used to generate the simulations. (ZIP) [file pone.0262749.s001.zip › S1_File/RADIUS/models/HEALTHY_HUMAN_REAL_ANKLE/docs/RADIUS_UserGuide_SevenLinkAmputee.pdf]

User Guide for **R**obot and **A**mputee **D**ynam**I**cs **U**nderactuated **S**imulation (RADIUS) with Details on the SEVEN_LINK_AMPUTEE Model

Anne E. Martin

August 5, 2014

1 Introduction

This guide describes how to use and modify RADIUS with the SEVEN_LINK_AMPUTEE model. Most of the information in Sec. 2 is applicable to all models.

RADIUS is a simulation and optimization platform for planer, underactuated bipeds. It also provides plotting and animation capabilities.

2 Using RADIUS

2.1 Installation

This section describes how to set RADIUS up on a computer for the first time.

1. Make sure MATLAB is installed on the computer.

2. Copy RADIUS onto your computer. Note that the file structure is very important. It is described in Sec. **??**. Create (or copy) the head folder RADIUS. The critical file to copy is r.m and the critical folders to copy are

   - MATLAB and all subfolders, and
   - models/<desired models>/MATLAB and all subfolders.

   To perform simulation, the folder models/<desired models>/gaitFiles must exist with at least one gait file in it. It is recommended, but not required to copy models/<desired models>/docs. As might be expected, the folder models/<desired models>/simData contains saved simulations, the folder models/<desired models>/video contains saved animations, and the folder models/<desired models>/plots contains saved plots.

2.2 Start-Up

This section provides a detailed description of how to start and initialize the program.

1. Start MATLAB and change to the folder RADIUS.

2. Type r at the command line prompt. If the current model has a lot of subfolders, it can take a long time for the program to start.

3. If the current model is the desired model, RADIUS is initialized. Otherwise, type L to load the model and choose the desired model from the list. Wait for the model to load. It can take a long time. Once the model has loaded, RADIUS is initialized.

To change the model at any point, repeat step 3.

2.3 Using the Program

2.3.1 Simulate (S)

Use this option to simulate a gait. The GUI allows you to choose the number of steps to simulate and whether or not to save the simulation. It then allows you to choose the gait to simulate. If saved, it is saved in models/<current model>/simData/<subfolder>/<name gait file>_<num steps>steps.mat where <subfolder> and <name gait file> are determined from the selected gait file. Details on what must be included in the gait file can be found in Sec. 2.4. Details on the struct that is generated can be found in Sec. 2.5.

2.3.2 Optimize (O)

Use this option to optimize a gait with respect to some user-selected objective function. After selecting this option, you choose the gait to optimize (the seed gait) and a GUI with optimization options appears. Make any desired changes on the GUI and then press OK. The optimization will run and save the optimized gait in models/<current model>/gaitFiles/<subfolder>/param_set_<k>.m where <subfolder> is the subfolder that the seed gait is in and <k> is smallest positive integer such that the new gait file will not overwrite any existing files. If desired, a simulation will be run for the number of steps specified in icData and saved in models/<current model>/simData/<subfolder>/param_set_<k>_<num steps>steps.mat. Details on what must be included in the gait file can be found in Sec. 2.4. Details on the struct that is generated can be found in Sec. 2.5.

2.3.3 Animate (A)

Selecting this option launches the animation GUI. Use the top pane to choose the desired options

1. Scroll Animation: If this is checked and the simulation has more than three steps, the animation is zoomed in and moves with the biped. This is good for animating long sequences of steps.

2. Plot Joint Angles: If this is checked, the joint angles are plotted under the animation with a moving star indicating the current set of angles.

3. Plot Joint Velocities: If this is checked, the joint angular velocities are plotted under the animation with a moving star indicating the current set of velocities.

4. Wait to Start: If this is checked, all of the set up is done but the animation doesn't start until a key is pressed.

5. Save Animation: If this option is checked, the animation is saved in models/<current model>/video/<sim file>.<ext> where <sim file> is the name of the simulation file and may include subdirectories. Use the dropdown box to chose the format to save the movie in. Note that when saving, the speed of the animation will typically be slower than the speed of the saved movie.

The bottom pane offers speed control. 1 is fastest, 0 is slowest. Note that the actual permitted interval is (0,1). For saved movies, setting speed = 0.5 plays at normal speed, speed = 0.25 plays at half speed, and speed = 0.5 plays at double speed. Pressing either of the buttons starts the animation.

- Choose Simulation: Press this button to animate a simulation that has already been saved.
- Create Simulation: Press this button to create a new simulation and then animate it. It is equivalent to first using the simulate options on the main menu and then animating using the choose simulation button.

2.3.4 Plot (P)

Selecting this option launches the plotting GUI (Fig. 1). The plots are generated using simulation files (Section 2.5). For the amputee models, the stance phase for both the amputated and contralateral sides is plotted first, followed by the swing phase. For the contralateral side, this means that the data from the simulation is reordered. Use the plots box to choose what to plot. For the SEVEN_LINK_AMPUTEE model, the options are:

- Phase Variable: Plots the phase variables given in the fields s_P and s_C.
- Joint Angles: Plots the joint angles in the **biomechanics** (not model) convention (Fig. 2, Section 3.1). Use the radio buttons to choose if the angles are plotted in degrees or radians.
- Angular Velocity: Plots the joint angular velocities using the biomechanics convention in rad/s.
- Angular Acceleration: Plots the joint angular accelerations using the biomechanics convention in rad/s^2.
- Joint Torques: Plots the joint torques using the model convention (Section 3.2).
- Ground Reaction Forces: Plots the ground reaction forces in the x and y directions.
- Socket Interaction Forces: Plots the socket interaction forces F_x, F_y, and F_M (Fig. 5).
- Swing Foot Height: Plots the height of the swing foot.

Use the x-axis box to choose what the x-axis is. For the SEVEN_LINK_AMPUTEE model, the options are:

- Time: Use time directly (units of seconds).
- % gait: Normalize time so that each step has a duration of 1 (dimensionless). This is what is typically done in the biomechanics literature.
- Phase - Loop: Use the phase variables s_P (for the prosthesis data) and s_C (for the human data). Because these values reset at the start of each step, multiple steps will be plotted on top of each other.

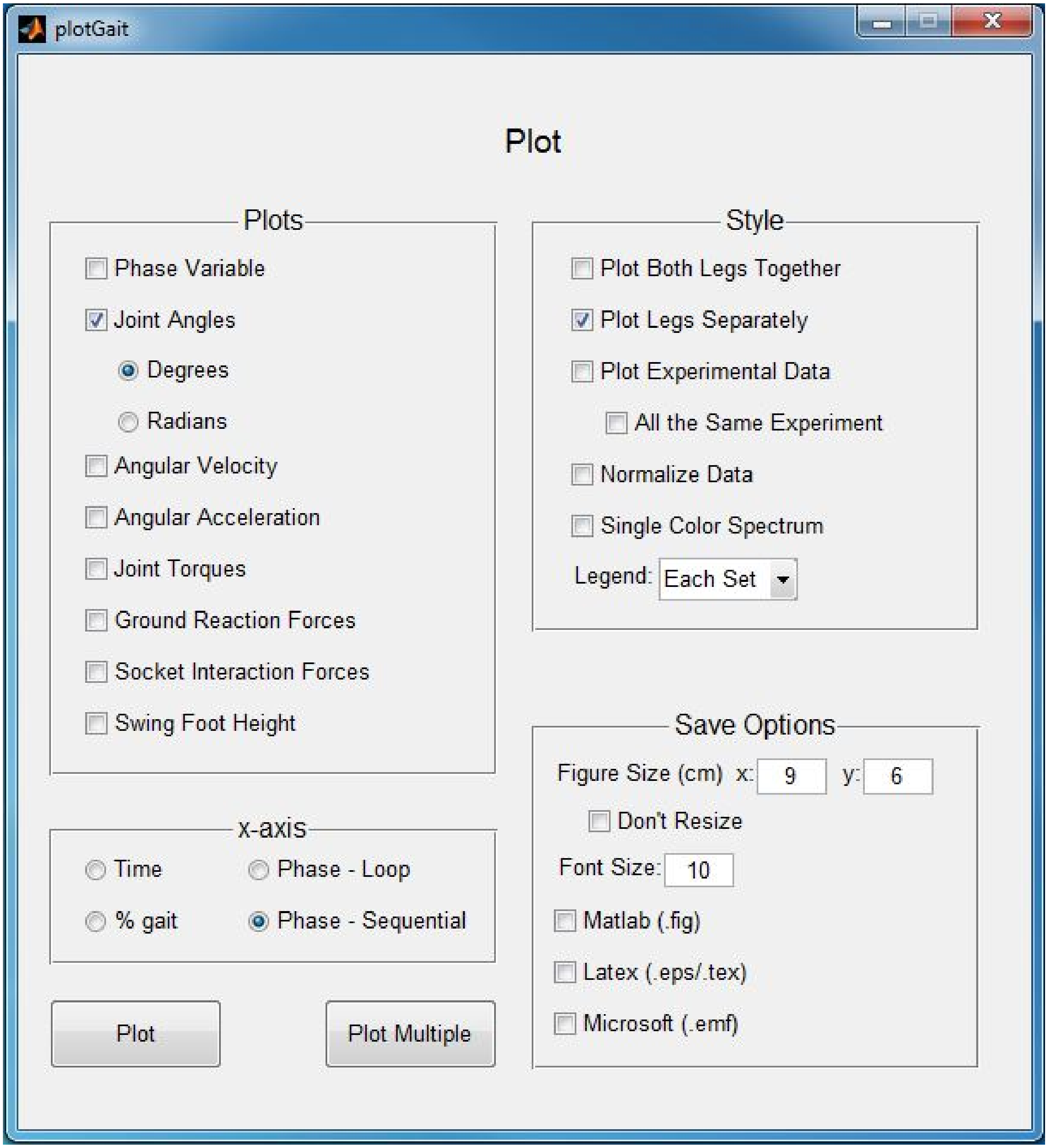

Figure 1: The plotting GUI for the SEVEN_LINK_AMPUTEE model.

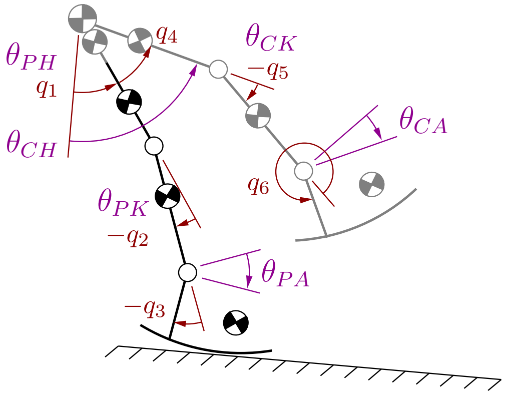

Figure 2: The angles for the SEVEN_LINK_AMPUTEE model. The angles used for simulation and optimization are indicated with the q_i's. The biomechanics convention used for plotting is indicated with the θ_i's. See Section 3.1 for details.

- Phase - Sequential: Use the phase variables s_P (for the prosthesis data) and s_C (for the human data). To prevent multiple steps being plotted on top of each other, the axis value is shifted by the axis value at the end of the previous step.

The style box is used to control various aspects of the plots. For the SEVEN_LINK_AMPUTEE model, the options are:

- Plot Both Legs Together: Plots both the amputated and contralateral data on the same set of axes. This is useful for comparing how similar the amputated and contralateral data is.

- Plot Legs Separately: Plots the amputated and contralateral data on separate axes. This is useful for generating plots that are not as cluttered. If both 'Plot Both Legs Together' and 'Plot Legs Separately' are unchecked, the data will be plotted as if 'Plot Legs Separately' were checked.

- Plot Experimental Data: In addition to the simulation data, also plot experimental data. The experimental data must stored as a struct with the same format as the simulation struct, and it must contain all of the fields needed for plotting with desired options. If the experimental data does not have all of the fields that the simulation does (likely), be sure those plots are unchecked in the plots box.

- All the Same Experiment: If checked, only plot one experimental gait. This is useful for comparing multiple simulations to an experiment.

- Normalize Data: If checked, much of the data is nondimensionalized. Specifically, all torques are divided by leg length $\times$ total mass $\times$ gravitational acceleration, all forces are divided by total mass $\times$ gravitational acceleration, and all lengths are divided by leg length [1].

- Single Color Spectrum: Normally, each gait gets a distinct color. However, if this box is checked, the gaits are plotted using grayscale. This is convenient for generating figures for papers.

- Use this to choose how many plots get legends
  - All: Every plot gets a legend.
  - Each Set: The first plot for each of the different plot types (i.e. for each option in the 'Plots' box) receives a legend. Plots for 'Plot Both Legs Together' and 'Plot Legs Separately' are considered separate (i.e. if both are checked, the first plot for each will receive a legend).
  - None: No plot gets a legend.

The Save Options box is used to control how the figures get saved. All figures are saved in `models/<current model>/plots`. More details on saving can be found in `Presentation Format Users Guide.pdf`. If the Plot button is pressed, the name of the figure is the name of the simulation file with an identifier appended to the end. If the Plot Multiple button is pressed, the user provides the base name of the files. The options are:

- Figure Size: The desired size of the figure in cm. If the figure should not be resized, check the don't resize box. Note that these values are only used if the figures are saved for Latex or Microsoft. For papers, save the figure at approximately the correct size so that the font is readable. For a two-column paper with a one-column figure, 9×6 cm is generally about the correct size. For a two-column figure, 18×8 cm is generally about the correct size.

- Font Size: The size of the font. Note that this value is only used if the figures are saved for Latex or Microsoft.

- Matlab: saves fig files for all plots

- Latex: saves eps and tex files for all plots for use in LaTeX. To insert a figure into a LaTeXdocument, use the package pstool and compile using pdflatex with the option `-shell-escape`. A minimal example is shown below

```
\documentclass{article}
\usepackage{pstool} % Load the package
\begin{document}
\psfragfig{myfig} % Put the graphic back together
\resizebox{width}{height}{\psfragfig{myfig}} % Put the graphic
    back together and adjusts the sizing. To keep the aspect
    ratio, specify either width or height, and set the other
    value as !.
\end{document}
```

- Microsoft: saves an emf file for all plots for use in Microsoft Office programs.

2.4 Gait Files

All of the information needed for simulation and optimization are contained in the gait file. The gait files function much like header files in that they contain the actual numbers used to define both the model and the controller. Each gait has its own gait file. The gait file is a struct that has been written out as a function. For simulation, the required fields are

- modelParams: all of the information about link lengths and masses and other quantities of that nature. For the SEVEN_LINK_AMPUTEE model, see Table 1 for a list of the required quantities.
- ctrlParams: all of the information needed to control the biped. For the SEVEN_LINK_AMPUTEE model, there are four required subfields
  - calculateHumanInput: a handle to the MATLAB function that calculates the joint torques for the human portion of the model.
  - calculateProsthesisInput: a handle to the MATLAB function that calculates the motor torques for the prosthesis.
  - calculateHumanPhaseVariable: a handle to the MATLAB function the calculates the phase variable for the human controller.
  - calculateProsthesisPhaseVariable: a handle to the MATLAB function the calculates the phase variable for the prosthesis controller.

  These functions should be located in `models/SEVEN_LINK_AMPUTEE/MATLAB/controlFunctions`. Other fields should be included as needed for the controllers to work properly. To change the way a biped walks without changing the form of the controller, simply change the values of the control fields. For example, if the controller is a feedback linearizing controller with the output encoded as a Bézeir polynomial, changing the terms in the alpha field changes the way the biped walks. To change the form of a controller, simply write the new function and change the handle in the gait file. See Sec. 3.2 for details on what is expected.
- integratorSettings: these are passed directly to `ode45()`, so see the MATLAB documentation for explanations. The required subfields are
  - AbsTol
  - RelTol
- icData: information about the initial conditions. The required subfields are
  - numSteps: the default number of steps to simulate. This is not used for the simulation option directly off of the menu, but it is used when the simulation function is called by other functions.
  - x0: the initial state of the biped. Positions are first, then velocities.

For optimization, in addition to the above fields, three more fields are required.

- objParams: this is an array of structs that is used to specify the objective function. Each possible objective function requires a series of two sequential structs. The first struct in each pair (all of the odd-indexed structs) are used to specify which objective function to use. The second struct in each pair (all of the even-indexed structs) specifies the function handle for the objective function. This somewhat convoluted method is required so that the GUI can be built programmatically using the gait file. An example is provided below.

1. `c2=c2+1;` This is a counter used to index the array. For the first objective function in the file, replace with `c2=1;`
2. `OUT.objParams(c2).field = '<name of function>';` What is in this field does not really matter, but it is suggested to use the name of the objective function. However, all of the `field` fields in the array of structs must be unique (see below for why).
3. `OUT.objParams(c2).value = <true or false>;` If true, use this objective function in the optimization. Only one of the odd-indexed structs can have a value of true. All others must be false.
4. `OUT.objParams(c2).parent = c2;` This field is used to group items together in the GUI.
5. `c2=c2+1;` Increment the counter.
6. `OUT.objParams(c2).field = '<name of function>_function';` Again, what is in this field does not really matter, but it is suggested to use the name of the objective function with `_function` appended to the end.
7. `OUT.objParams(c2).value = '<name of function>';` This field must contain the name of the objective function, which must match the name of the file that defines the function. The objective functions should be kept in the folder `models/<current model>/MATLAB/optimizationFunctions/objectiveFunctions`.
8. `OUT.objParams(c2).parent = c2-1;` This struct is grouped with the previous struct.

The optimization will automatically convert the array of structs (objParams) into a normal struct (objStruct) in which the names of the objStruct fields are given by the `field` fields of objParams and the values of the objStruct fields are given by the `value` fields of objParams. For the example, objStruct is given by

- `objStruct.<name of function> = <true or false>`
- `objStruct.<name of function>_function = <name of function>`

- constrParams: this is an array of structs used to specify the constraints for the optimization. To allow the GUI to be built, an array of structs must be used. However, the optimization automatically converts the array of structs (constrParams) into a normal struct (constrStruct) for easier use. Each struct in constrParams must contain the following fields
  - field: the name of the field in constrStruct
  - value: the value, it may be a boolean (true/false), a number, or a string.
  - boolean: if equal to 1, the GUI uses a checkbox, otherwise the GUI uses a textbox.
  - parent: the index of the parent item. The parent item must always be a boolean and is used to turn constraints on and off. The children fields provide information needed to calculate the constraint(s).

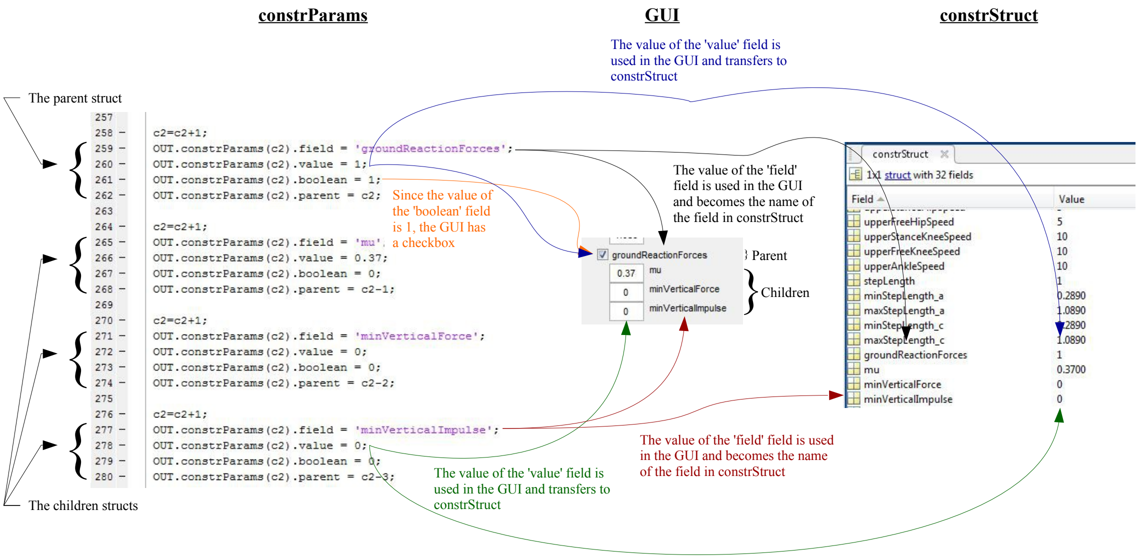

Figure 3: An example of how quantities transfer from constrParams to the GUI and constrStruct.

After conversion into a normal struct, the fields of constrStruct are the values of the `field` fields of constrParams, and the values of the fields are the values of the `value` fields of constrParams (Fig. 3).

- fminconParams: an array of structs that provides information for the optimization. Similar to objParams and constrParams, fminconParams is transformed into a normal struct named fminconStruct in the optimization. The fields for fminconParams are
  - field: the name of the fields for fminconStruct
  - value: the value of the fields for fminconStruct
  - boolean: if true, the GUI uses a checkbox for this item, otherwise it uses a textbox.

  fminconStruct must have the following fields (i.e. there must be a struct in fminconParams in which the `field` field has the following values)

  - evaluateGait: a function handle to a function that runs a simulation, calculates the value of the objective function, and calculates the nonlinear constraints.
  - dosort: a function handle to a function that sorts modelParams and ctrlParams into variables the optimization can directly change (the optimization variables) and variables that it cannot.
  - unsort: a function handle to a function that rearranges the results of dosort back into modelParams and ctrlParams.
  - boundType: To help the optimization run better, there are bounds on how much the optimization variables can change. These bounds are symmetric about the initial value of the optimization variables. The options are

  * offset: bound = val $\pm$ paramFreedom
  * scale: bound = val(1 $\pm$ paramFreedom)
  * both: the larger of the two options
- paramFreedom: how large the bound on the optimization variables is.
- runSim: if true and the optimization converged, runs and saves the simulation after the optimization completes.
- alwaysRunSim: if true, runs the simulation regardless of if the optimization converges.
- correctIC: the optimization only finds approximately periodic gaits because it greatly speeds up the optimization and increases the likelihood of convergence [2]. If correctIC is true, after the optimization has completed, the actual periodic gait is found.
- correctIC_fun: The function handle to the function that finds the periodic gait. If correctIC is false, it is acceptable to use and empty string for the value of correctIC_fun.
- DiffMinChange: see MATLAB documentation
- DiffMaxChange: see MATLAB documentation
- Display: see MATLAB documentation
- MaxFunEvals: see MATLAB documentation
- MaxIter: see MATLAB documentation
- MaxSQPIter: see MATLAB documentation
- TolCon: see MATLAB documentation
- TolFun: see MATLAB documentation
- TolX: see MATLAB documentation
- RelLineSrchBnd: see MATLAB documentation. If not used, use an empty string (`''`) rather than empty braces so that the GUI builds correctly.
- UseParallel: see MATLAB documentation

2.5 Simulation Files

After a simulation is run, the results of the simulation are stored in a struct called out. If the simulation is saved, the `.mat` file that is saved contains out. Assume the simulation took M steps requiring a total of n timesteps. Also assume the model has N links. The following details the fields in out:

- runDate: a timestamp of when the simulation was run
- t: an $1 \times n$ vector of timepoints
- x: a $2N \times n$ matrix of generalized coordinates and velocities. The top N rows are the generalized coordinates and the bottom N rows are the generalized velocities.
- eventFlag: an $1 \times M$ vector containing flags that indicate how each step ended. A value of 1 indicates a successful step while other values indicate failure.

- eventIndex: an $1 \times (M + 1)$ vector containing the index for the instant before each impact. To access all of the values for step j, use `eventIndex(j)+1:eventIndex(j+1)`.

- output: A struct containing additional information about the gait. Exactly what is saved depends on the model (it is specified using the function `simOutput`). For the SEVEN_LINK_AMPUTEE model, the fields are

  - aveSpeed: an $1 \times M$ vector containing the speed of each step which is equal to (step length)/(step duration).
  - stepLengths: an $1 \times M$ vector containing the length of each step
  - stepDurations: an $1 \times M$ vector containing the duration of each step
  - stanceSide: an $1 \times n$ vector containing flags to indicate which leg is in stance
    - 0 = amputated
    - 1 = healthy
  - sP: an $1 \times n$ vector containing the phase variable for the prosthesis, normalized to remain between 0 and 1
  - sH: an $1 \times n$ vector containing the phase variable for the human, normalized to remain between 0 and 1
  - q: a $6 \times n$ matrix of joint angles $[q_1\ q_2\ q_3\ q_4\ q_5\ q_6]^T$
  - dq: a $6 \times n$ matrix of joint angular velocities
  - ddq: a $6 \times n$ matrix of joint angular accelerations
  - p: a $2 \times n$ matrix of the position of the hip $[p_x\ p_y]^T$
  - dp: a $2 \times n$ matrix of hip velocities
  - ddp: a $2 \times n$ matrix of hip accelerations
  - qe: an $8 \times n$ matrix of extended generalized coordinates $[q_1\ q_2\ q_3\ q_4\ q_5\ q_6\ p_x\ p_y]^T$
  - dqe: an $8 \times n$ matrix of the extended generalized coordinate velocities
  - ddqe: an $8 \times n$ matrix of the extended generalized coordinate accelerations
  - qp: a $5 \times n$ matrix of prostheses generalized coordinates $[q_1\ q_2\ q_3\ p_x\ p_y]^T$
  - dqp: a $5 \times n$ matrix of the prostheses generalized coordinate velocities
  - ddqp: a $5 \times n$ matrix of the prostheses generalized coordinate accelerations
  - swingFootDisplacement: an $1 \times n$ vector containing the horizontal position of the lowest point on the swing foot relative to the initial point of contact of the stance foot
  - swingFootHeight: an $1 \times n$ vector containing the height of the lowest point on the swing foot
  - uP: a $2 \times n$ matrix containing the motor torques for the prosthesis $[u_2\ u_3]^T$
  - uH: a $3 \times n$ matrix containing the joint torques for the human $[u_4\ u_5\ u_6]^T$
  - F: a $3 \times n$ matrix containing the socket interaction forces $[F_x\ F_y\ F_M]^T$

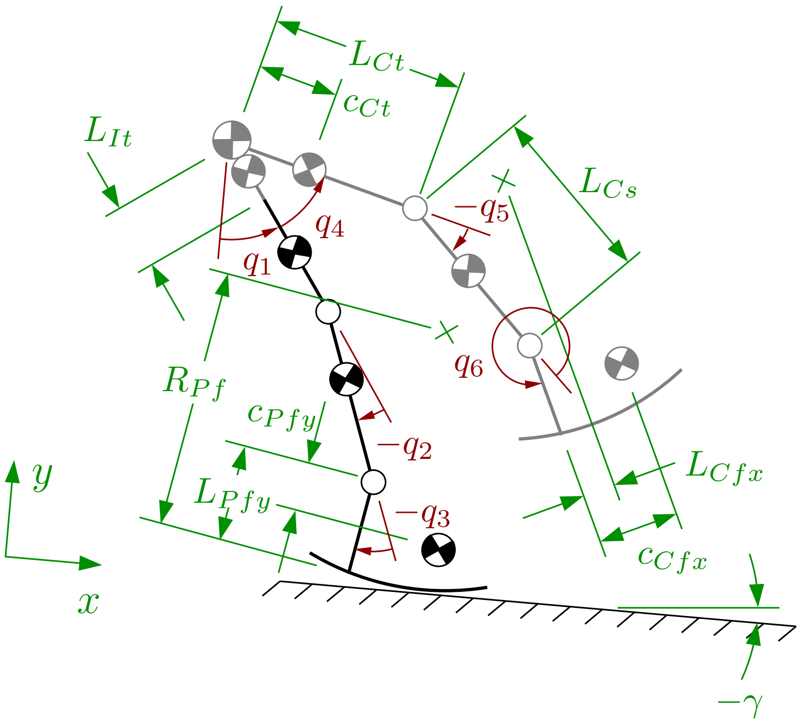

Figure 4: Schematic of the amputee model. The prosthesis is shown in black and the human is shown in gray. Joint angles are shown in red. Constant parameters are indicated with green. For clarity, not all parameters are shown.

  - G: a $2 \times n$ matrix containing the ground reaction force $[G_x \ G_y]^T$
  - g: a $2 \times M$ matrix containing the impulsive ground reaction force at impact $[g_x \ g_y]^T$

- modelParams: a copy of the modelParams struct from the gait file
- ctrlParams: a copy of the ctrlParams struct from the gait file
- extraData: a copy of the whole struct in the the gait file

3 Seven-Link Amputee Model

The model consists of seven links plus a point mass at the hip to represent the upper body. In Fig. 4, the prosthesis is shown in black and the human is shown in gray. The prosthesis consists of a thigh, shank, and foot. The human consists of a residual thigh on the amputated thigh, a thigh, shank, and foot on the contralateral side, and a point mass at the hip. The biped walks from left to right in the positive x-direction. It is assumed to roll without slip.

3.1 Joint Angles

The x-axis of the global reference frame is aligned with the walking surface. The biped is assumed to walk in the positive x direction. The y-axis points up from the walking surface and is perpendicular

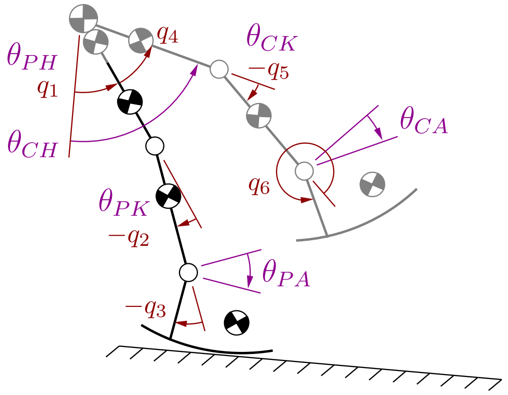

Figure 2 repeated: The angles for the SEVEN_LINK_AMPUTEE model. The angles used for simulation and optimization are indicated with the q_i's. The biomechanics convention used for plotting is indicated with the θ_i's.

to it. At the start of each step, the origin of the global reference frame is reset to the point of contact of the stance foot.

All angles are measured counter-clockwise (CCW) positive. Angles q_1-q_3 are prosthesis angles, and angles q_1, q_4-q_6 are human angles. q_1 is measured from the perpendicular of the walking surface to the prosthetic thigh. This is the absolute angle of both the prosthetic thigh and the residual thigh. q_2 is measured from the prosthetic thigh to the prosthetic shank. q_3 is measured from the prosthetic shank to the prosthetic foot. q_4 is measured from the prosthetic thigh to the contralateral thigh. q_5 is measured from the contralateral thigh to the contralateral shank. q_6 is measured from the contralateral shank to the contralateral foot. The position of the hip is given by (p_x, p_y). Due to the resetting of the global reference frame, p_x is not continuous between steps.

There are three sets of generalized coordinates used.

- The joint angles are given by $q = [q_1\ q_2\ q_3\ q_4\ q_5\ q_6]^T$
- The extended generalized coordinates are given by $q_e = [q_1\ q_2\ q_3\ q_4\ q_5\ q_6\ p_x\ p_y]^T$
- The prosthesis generalized coordinates are given by $q_p = [q_1\ q_2\ q_3\ p_x\ p_y]^T$

For plotting, the model angles are transformed into a more conventional biomechanics format (Fig. 2). The prosthetic hip angle θ_{PH} is identical to q_1. That is, it is measured CCW positive from the perpendicular of the walking surface to the prosthetic thigh. The contralateral hip angle θ_{CH} is measured CCW positive from the perpendicular of the walking surface to the contralateral thigh. The prosthetic knee angle θ_{PK} is measured clockwise (CW) positive from the prosthetic thigh to the prosthetic shank. The contralateral knee angle θ_{CK} is measured CW positive from the contralateral thigh to the contralateral shank. The prosthetic ankle angle θ_{PA} is measured CW positive from the perpendicular of the prosthetic shank to the perpendicular of the prosthetic foot. The contralateral ankle angle θ_{CA} is measured CW positive from the perpendicular of the contralateral shank to the perpendicular of the contralateral foot. To convert between the conventions, use the following

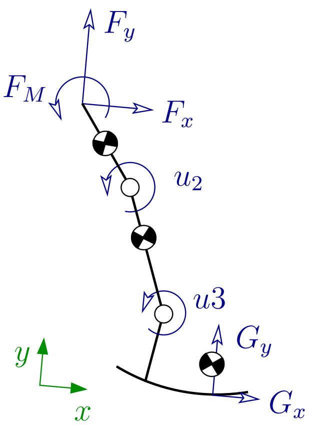

Figure 5: External forces acting on the prosthesis during stance.

formulas

$$\theta_{PH} = q_1 \tag{1}$$
$$\theta_{CH} = q_1 + q_4 \tag{2}$$
$$\theta_{PK} = -q_2 \tag{3}$$
$$\theta_{CK} = -q_5 \tag{4}$$
$$\theta_{PA} = -q_3 \tag{5}$$
$$\theta_{CA} = -q_6 \tag{6}$$

3.2 External Forces

A CCW positive torque u can be applied at joints 2 through 6. q_1 is unactuated. The interaction between the human and the prosthesis is captured in the socket interaction force F (Fig. 5). Considering the prosthesis, the forces are positive in the positive x- and y-directions, and the moment is positive CCW. The leg that is in stance also experiences horizontal and vertical ground reaction force G (GRF). There is no moment at the foot-ground interface due to the curved foot.

It is assumed that the prosthesis only knows information about itself. In other words, it knows the prosthesis generalized coordinates and associated derivatives, the socket interaction force, and the ground reaction force on the prosthetic side. The prosthesis has no information about what the human joints are doing. On the other hand, it is assumed the human has complete information about itself plus the prosthesis. To do feedback linearization on both the prosthesis and the human, it can easily be shown (Sec. 3.5) that the human joint torques, the prosthesis motor torques, and the socket iteration force are related. The torques can be written as

$$u_P = \widehat{\beta}_P + \overline{\beta}_P \cdot F \tag{7}$$
$$u_H = \widehat{\beta}_H + \widetilde{\beta}_H \cdot u_P \tag{8}$$

where the β_P's depend on the prosthesis generalized coordinates and the prosthesis output functions and the β_H's depend on the extended generalized coordinates and the human output functions. In

the code, the function specified by `ctrlParams.calculateProsthesisInput` in the gait file must return $\widehat{\beta}_P$ (a 2×1 matrix) and $\overline{\beta}_P$ (a 2×3 matrix). Either matrix may be the zero matrix. Similarly, the function specified by `ctrlParams.calculateHumanInput` must return $\widehat{\beta}_H$ (a 3×1 matrix) and $\widetilde{\beta}_H$ (a 3×2 matrix).

3.3 Constant Parameters

To identify link lengths and masses, the following scheme is used:

- The first letter indicates which quantity
  - Length of segment (L)
  - Center of mass (CoM) location measured from the proximal joint or the human prosthesis interface (c)
  - Radius of foot (R)
  - Mass of segment (M)
  - Inertia of segment measured about the segment's CoM (J)
- The first subscript indicates which side
  - Prosthesis (P)
  - Contralateral side (C)
  - Intact portion of the limb on the amputated side, i.e. the residual limb (I)
  - Composite residual limb-prosthetic limb for the amputated side (A)
- The second subscript indicates which body part
  - Hip (h)
  - Thigh (t)
  - Shank (s)
  - Foot (f)
- The third subscript indicates which direction (if needed)
  - Anterior/posterior (x)
  - Proximal/distal (y)

Table 1: List of all model parameters used

Quantity	Symbol	Code Name
Mass of hip	M_{Ch}	MCh

Continued on next page

Table 1 – continued from previous page

Quantity	Symbol	Code Name
Length of residual thigh	L_{It}	LIt
CoM location of residual thigh measured from the hip	c_{It}	cIt
Mass of residual thigh	M_{It}	MIt
Inertia of the residual thigh measured about the CoM	J_{It}	JIt
Length of contralateral thigh	L_{Ct}	LCt
CoM location of the contralateral thigh measured from the hip	c_{Ct}	cCt
Mass of the contralateral thigh	M_{Ct}	MCt
Inertia of the contralateral thigh measured about the CoM	J_{Ct}	JCt
Length of the contralateral shank	L_{Cs}	LCs
CoM location of the contralateral shank measured from the knee	c_{Cs}	cCs
Mass of the contralateral shank	M_{Cs}	MCs
Inertia of the contralateral shank measured about the CoM	J_{Cs}	JCs
Radius of the contralateral foot	R_{Cf}	RCf
Vertical distance from the lowest point on the contralateral foot to the contralateral ankle joint when	L_{Cfy}	LCfy
Horizontal distance from the contralateral ankle joint to the contralateral foot center of curvature when	L_{Cfx}	LCfx
Vertical distance from the contralateral ankle joint to the contralateral foot CoM when	c_{Cfy}	cCfy
Horizontal distance from the contralateral ankle joint to the contralateral foot CoM when	c_{Cfx}	cCfx
Mass of the contralateral foot	M_{Cf}	MCf
The inertia of the contralateral foot is neglected	0	0
Length of prosthetic thigh	L_{Pt}	LPt
CoM location of the prosthetic thigh measured from the human-prosthesis interface	c_{Pt}	cPt
Mass of the prosthetic thigh	M_{Pt}	MPt
Inertia of the prosthetic thigh measured about the CoM	J_{Pt}	JPt
Length of the prosthetic shank	L_{Ps}	LPs
CoM location of the prosthetic shank measured from the knee	c_{Ps}	cPs
Mass of the prosthetic shank	M_{Ps}	MPs
Inertia of the prosthetic shank measured about the CoM	J_{Ps}	JPs
Radius of the prosthetic foot	R_{Pf}	RPf

Continued on next page

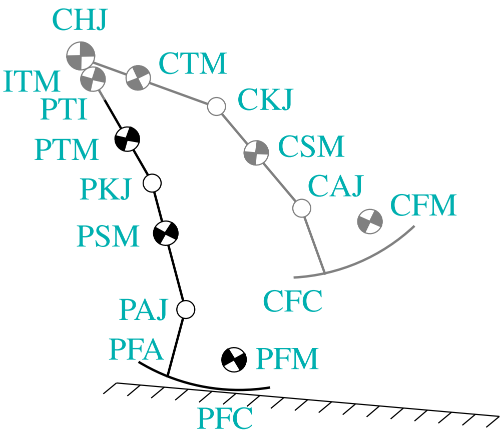

Figure 6: Schematic showing key positions on the biped. Not shown are the foot centers of curvature (PFR and CFR) and the composite residual thigh-prosthetic thigh CoM (ATM).

Table 1 – continued from previous page

**Quantity**	**Symbol**	**Code Name**
Vertical distance from the lowest point on the prosthetic foot to the prosthetic ankle joint when	L_{Pfy}	LPfy
Horizontal distance from the prosthetic ankle joint to the prosthetic foot center of curvature when	L_{Pfx}	LPfx
Vertical distance from the prosthetic ankle joint to the contralateral foot CoM when	c_{Pfy}	cPfy
Horizontal distance from the prosthetic ankle joint to the prosthetic foot CoM when	c_{Pfx}	cPfx
Mass of the prosthetic foot	M_{Pf}	MPf
The inertia of the prosthetic foot is neglected	0	0
The angle the ground makes with horizontal, measured CCW and in radians	γ	gamma
Gravitational acceleration (positive)	g	grav
Length of the combined residual thigh-prosthetic thigh (calculated)	L_{At}	LAt
CoM location the combined residual thigh-prosthetic thigh measured from the hip (calculated)	c_{At}	cAt
Mass of the combined residual thigh-prosthetic thigh (calculated)	M_{At}	MAt
Inertia of the combined residual thigh-prosthetic thigh measured about the CoM (calculated)	J_{At}	JAt

3.4 Key Positions

For deriving the equations of motion and for animation, there are several important points on the biped (Fig. 6). When naming these points, the following convention is used:

- First letter indicates which side
  - Prosthesis (P)
  - Contralateral side (C)
  - Intact portion of the limb on the amputated side, i.e. the residual limb (I)
  - Composite residual limb-prosthetic limb for the amputated side (A)
- The second letter indicates which body part or joint
  - Hip (H)
  - Knee (K)
  - Ankle (A)
  - Thigh (T)
  - Shank (S)
  - Foot (F)
- The third letter indicates what type of point
  - Joint (J)
  - CoM (M)
  - Contact point or lowest point on foot (C)
  - Center of curvature, i.e. the point where all radii meet (R)
  - Interface between prosthesis and human (I)
  - Attachment between upright portion of foot and curved portion of foot (A)

There are two points where multiple labels would be appropriate. These points are clarified below:

- The interface between the human and the prosthesis is indicated with PTI
- The hip joint and the position of the hip mass are located at the same point and indicated with CHJ

3.5 Derivation of Feedback Linearizing Inputs

The equations of motion (EoM) for the prosthesis can be written as

$$M_P\ddot{q}_P + C_P\dot{q}_P + N_P - E_P^T G = B_P u_P + J^T F \tag{9}$$

where $q_P = [q_1\ q_2\ q_3\ p_x\ p_y]^T$ is the prosthesis generalized coordinates, M_P is the 5×5 inertia matrix for the prosthesis, C_P is the 5×5 matrix containing the centripetal and Coriolis terms for the prosthesis, N_P is the 5×1 vector containing the gravity terms for the prosthesis, E_P is a 2×5 constraint matrix used to find the GRF, $G = [G_x\ G_y]^T$ is the GRF acting on the prosthesis (equal to zero when the prosthesis is in swing), B_P is the 5×2 matrix relating the prosthesis motor torques to the prosthesis generalized coordinates, $u_P = [u_2\ u_3]^T$ is the prosthesis motor torques, J is the Jacobian matrix relating the socket interaction forces to the prosthesis generalized coordinates, and $F = [F_x\ F_y\ F_M]^T$ is the socket interaction forces. When the prosthesis is in stance, the constraint equation can be used to attach the prosthesis to the ground.

$$E_P\dot{q}_P = 0. \tag{10}$$

This can be differentiated once with respect to time to obtain

$$\dot{E}_P\dot{q}_P + E_P\ddot{q}_P = 0. \tag{11}$$

Solving the EoM (Eq. 9) for $\ddot{q}_P$, substituting it into Eq. 11, and solving for G gives [3]

$$G = \widehat{\lambda} + \widetilde{\lambda} u_P + \overline{\lambda} F \tag{12}$$

where

$$\widehat{\lambda} = W(E_P M_P^{-1}(C_P\dot{q}_P + N_P) - \dot{E}_P\dot{q}_P) \tag{13}$$

$$\widetilde{\lambda} = -W E_P M_P^{-1} B_P \tag{14}$$

$$\overline{\lambda} = -W E_P M_P^{-1} J^T \tag{15}$$

$$W = (E_P M_P^{-1} E_P^T)^{-1}. \tag{16}$$

Substituting G (Eq. 12) back into the EoM (Eq. 9) and solving for $\ddot{q}_P$ gives

$$\ddot{q}_P = M_P^{-1}(-C_P\dot{q}_P - N_P + E_P^T(\widehat{\lambda} + \widetilde{\lambda} u_P + \overline{\lambda} F) + B_P u_P + J^T F). \tag{17}$$

Now, define an output function for the prosthesis as

$$y_P = h_P(q_P) = H_{0P} q_P - h_{dP}(s_P(\theta_P(q_P))) \tag{18}$$

where h_{dP} is a vector-valued function of length 2 representing the desired prosthesis joint angles,

$$H_{0P} = \begin{bmatrix} 0 & 1 & 0 & 0 & 0 \\ 0 & 0 & 1 & 0 & 0 \end{bmatrix}, \tag{19}$$

$$s_P = \frac{\theta_P - \theta_P^+}{\theta_P^- - \theta_P^+}, \tag{20}$$

$$\theta_P = c_P q_P, \tag{21}$$

θ_P^+ is the value of θ_P at the start of the step, θ_P^- is the value of θ_P at the end of the step, and c_P is a 1×5 vector of constants. Differentiating Eq. 18 twice gives

$$\ddot{y}_P = \frac{\partial h_P}{\partial \theta_P}\ddot{q}_P - a_P, \tag{22}$$

where

$$\frac{\partial h_P}{\partial \theta_P} = H_{0P} - \frac{\partial h_{dP}}{\partial s_P}\frac{1}{\theta_P^- - \theta_P^+}c_P, \tag{23}$$

$$a_P = \frac{\partial^2 h_{dP}}{\partial s_P^2}\frac{(c_P\dot{q}_P)^2}{(\theta_P^- - \theta_P^+)^2}. \tag{24}$$

To do feedback linearization, set $\ddot{y}_P = v$ and substitute the prosthesis EoM (Eq. 17) into Eq. 22.

$$v = \frac{\partial h_P}{\partial \theta_P}M_P^{-1}(-C_P\dot{q}_P - N_P + E_P^T(\widehat{\lambda} + \widetilde{\lambda}u_P + \overline{\lambda}F) + B_P u_P + J^T F) - a_P \tag{25}$$

$$= \widehat{L_f^2 h_{dP}} + (\widetilde{L_f^2 h_{dP}} + L_g L_f h_{dP})u_P + (\overline{L_f^2 h_{dP}} + L_j L_f h_{dP})F \tag{26}$$

where

$$\widehat{L_f^2 h_{dP}} = -\frac{\partial h_P}{\partial \theta_P}M_P^{-1}(C_P\dot{q}_P + N_P - E_P^T\widehat{\lambda}) - a_P \tag{27}$$

$$\widetilde{L_f^2 h_{dP}} = \frac{\partial h_P}{\partial \theta_P}M_P^{-1}E_P^T\widetilde{\lambda} \tag{28}$$

$$\overline{L_F^2 h_{dP}} = \frac{\partial h_P}{\partial \theta_P}M_P^{-1}E_P^T\overline{\lambda} \tag{29}$$

$$L_g L_f h_{dP} = \frac{\partial h_P}{\partial \theta_P}M_P^{-1}B_P \tag{30}$$

$$L_j Lf h_{dP} = \frac{\partial h_P}{\partial \theta_P}M_P^{-1}J^T. \tag{31}$$

Solving for u_P gives

$$u_P = \widehat{\beta}_P + \overline{\beta}_P F \tag{32}$$

where

$$\widehat{\beta}_P = (\widetilde{L_f^2 h_{dP}} + L_g L_f h_{dP})^{-1}(v - \widehat{L_f^2 h_{dP}}) \tag{33}$$

$$\overline{\beta}_P = -(\widetilde{L_f^2 h_{dP}} + L_g L_f h_{dP})^{-1}(\overline{L_F^2 h_{dP}} + L_j Lf h_{dP}). \tag{34}$$

Note that we do not know F.

We repeat the procedure using the full human + prosthesis system to obtain an equation for u_H. The EoM for the full system can be written as

$$M_F\ddot{q}_e + C_F\dot{q}_e + N_F - E_F^T G_F = B_{FP}u_P + B_{FH}u_H \tag{35}$$

where $q_e = [q_1\ q_2\ q_3\ q_4\ q_5\ q_6\ p_x\ p_y]^T$ is the extended generalized coordinates, M_F is the 8×8 inertia matrix for the full system, C_F is the 8×8 matrix containing the centripetal and Coriolis

terms for the full system, N_F is the 8×1 vector containing the gravity terms for the full system, E_F is a 2×8 constraint matrix used to find the GRF, $G_F = [G_x \ G_y]^T$ is the GRF acting on the full system (equal to G when the prosthesis is in stance), B_{FP} is the 8×2 matrix relating the prosthesis motor torques to the extended generalized coordinates, $u_P = [u_2 \ u_3]^T$ is the prosthesis motor torques, B_{FH} is the 8×3 matrix relating the human joint torques to the extended generalized coordinates, and $u_H = [u_4 \ u_5 \ u_6]^T$ is the human joint torques. The constraint equation can be used to attach the biped to the ground.

$$E_F \dot{q}_e = 0. \tag{36}$$

This can be differentiated once with respect to time to obtain

$$\dot{E}_F \dot{q}_e + E_F \ddot{q}_e = 0. \tag{37}$$

Solving the EoM (Eq. 35) for $\ddot{q}_e$, substituting it into Eq. 37, and solving for G_F gives [3]

$$G_F = \lambda_1 + \lambda_2 u_P + \lambda_3 u_H \tag{38}$$

where

$$\lambda_1 = W_F(E_F M_F^{-1}(C_F \dot{q}_e + N_F) - \dot{E}_F \dot{q}_e) \tag{39}$$
$$\lambda_2 = -W_F E_F M_F^{-1} B_{FP} \tag{40}$$
$$\lambda_3 = -W_F E_F M_F^{-1} B_{FH} \tag{41}$$
$$W_F = (E_F M_F^{-1} E_F^T)^{-1}. \tag{42}$$

Substituting G_F (Eq. 38) back into the EoM (Eq. 35) and solving for $\ddot{q}_e$ gives

$$\ddot{q}_e = M_F^{-1}(-C_F \dot{q}_e - N_F + E_F^T(\lambda_1 + \lambda_2 u_P + \lambda_3 u_H) + B_{FP} u_P + B_{FH} u_H). \tag{43}$$

Now, define an output function for the human as

$$y_H = h_H(q_e) = H_{0H} q_e - h_{dH}(s_H(\theta_H(q_e))) \tag{44}$$

where h_{dH} is a vector-valued function of length 3 representing the desired human joint angles,

$$H_{0H} = \begin{bmatrix} 0 & 0 & 0 & 1 & 0 & 0 & 0 & 0 \\ 0 & 0 & 0 & 0 & 1 & 0 & 0 & 0 \\ 0 & 0 & 0 & 0 & 0 & 1 & 0 & 0 \end{bmatrix}, \tag{45}$$
$$s_H = \frac{\theta_H - \theta_H^+}{\theta_H^- - \theta_H^+}, \tag{46}$$
$$\theta_H = c_H q_e, \tag{47}$$

θ_H^+ is the value of θ_H at the start of the step, θ_H^- is the value of θ_H at the end of the step, and c_H is a 1×8 vector of constants. Differentiating Eq. 44 twice gives

$$\ddot{y}_H = \frac{\partial h_H}{\partial \theta_H} \ddot{q}_H - a_H, \tag{48}$$

where

$$\frac{\partial h_H}{\partial \theta_H} = H_{0H} - \frac{\partial h_{dH}}{\partial s_H} \frac{1}{\theta_H^- - \theta_H^+} c_H, \tag{49}$$

$$a_H = \frac{\partial^2 h_{dH}}{\partial s_H^2} \frac{(c_H \dot{q}_H)^2}{(\theta_H^- - \theta_H^+)^2}. \tag{50}$$

To do feedback linearization, set $\ddot{y}_H = v_H$ and substitute the full EoM (Eq. 43) into Eq. 48.

$$v_H = \frac{\partial h_F}{\partial \theta_H} M_F^{-1}(-C_F \dot{q}_e - N_F + E_F^T(\lambda_1 + \lambda_2 u_P + \lambda_3 u_H) + B_{FP} u_P + B_{FH} u_H) - a_H \tag{51}$$

$$= L_f^2 h_{dH1} + (L_f^2 h_{dH2} + L_{g_P} L_f h_{dH}) u_P + (L_f^2 h_{dH3} + L_{g_H} L_f h_{dH}) u_H \tag{52}$$

where

$$L_f^2 h_{dH1} = -\frac{\partial h_H}{\partial \theta_H} M_F^{-1}(C_F \dot{q}_e + N_F - E_F^T \lambda_1) - a_H \tag{53}$$

$$L_f^2 h_{dH2} = \frac{\partial h_H}{\partial \theta_H} M_F^{-1} E_F^T \lambda_2 \tag{54}$$

$$L_F^2 h_{dH3} = \frac{\partial h_H}{\partial \theta_H} M_F^{-1} E_F^T \lambda_3 \tag{55}$$

$$L_{g_P} L_f h_{dH} = \frac{\partial h_H}{\partial \theta_H} M_F^{-1} B_{FP} \tag{56}$$

$$L_{g_H} L f h_{dH} = \frac{\partial h_H}{\partial \theta_H} M_F^{-1} B_{FH}. \tag{57}$$

Solving for u_H gives

$$u_H = \widehat{\beta}_H + \widetilde{\beta}_H u_P \tag{58}$$

where

$$\widehat{\beta}_H = (L_f^2 h_{dH3} + L_{g_H} L_f h_{dH})^{-1}(v_H - L_f^2 h_{dH1}) \tag{59}$$

$$\widetilde{\beta}_H = -(L_f^2 h_{dH3} + L_{g_H} L_f h_{dH})^{-1}(L_F^2 h_{dH2} + L_{g_P} L f h_{dH}). \tag{60}$$

Since we know that the motion of the prosthesis generalized coordinates must equal the motion of the equivalent generalized coordinates in the full system, we match the accelerations.

$$\ddot{q}_P = S \ddot{q}_e \tag{61}$$

where

$$S = \begin{bmatrix} 1 & 0 & 0 & 0 & 0 & 0 & 0 & 0 \\ 0 & 1 & 0 & 0 & 0 & 0 & 0 & 0 \\ 0 & 0 & 1 & 0 & 0 & 0 & 0 & 0 \\ 0 & 0 & 0 & 0 & 0 & 0 & 1 & 0 \\ 0 & 0 & 0 & 0 & 0 & 0 & 0 & 1 \end{bmatrix}. \tag{62}$$

Substituting in the EoM for the prosthesis (Eq. 17) and full system (Eq. 43) and the torques (Eqs. 32 and 58) and rearranging gives

$$F_{den} F = F_{num} \tag{63}$$

where

$$
\begin{aligned}
F_{den} =& M_P^{-1}E_P^T(\widetilde{\lambda}\overline{\beta}_P + \overline{\lambda}) + M_P^{-1}B_P\overline{\beta}_P + M_P^{-1}J^T - SM_F^{-1}E_F^T(\lambda_2\overline{\beta}_P + \lambda_3\widetilde{\beta}_H\overline{\beta}_P) && (64)\\
& - SM_F^{-1}B_{FP}\overline{\beta}_P - SM_F^{-1}B_{FH}\widetilde{\beta}_H\overline{\beta}_P, && (65)\\
F_{num} =& M_P^{-1}(C_P\dot{q}_P + N_P) - M_P^{-1}E_P^T(\widehat{\lambda} + \widetilde{\lambda}\widehat{\beta}_P) - M_P^{-1}B_P\widehat{\beta}_P - SM_F^{-1}(C_F\dot{q}_e + N_F) && (66)\\
& + SM_F^{-1}E_F^T(\lambda_1 + \lambda_2\widehat{\beta}_P + \lambda_3\widehat{\beta}_H + \lambda_3\widetilde{\beta}_H\widehat{\beta}_P) + SM_F^{-1}B_{FP}\widehat{\beta}_P && (67)\\
& + SM_F^{-1}B_{FH}(\widehat{\beta}_H + \widetilde{\beta}_H\widehat{\beta}_P). && (68)
\end{aligned}
$$

Since F_{den} is not square, F can be found using

$$F = (F_{den}^T F_{den})^{-1} F_{den}^T F_{num}. \quad (69)$$

The prosthesis motor torques can be found by substituting Eq. 69 into Eq. 32. The human joint torques can be found be substituting the prosthesis motor torques (Eq. 32) into Eq. 58.

References

[1] At L. Hof. Scaling gait data to body size. *Gait & Posture*, 4(3):222–3, May 1996.

[2] Anne E. Martin. *Predictive modeling of healthy and amputee walking using a simple planar model*. PhD thesis, University of Notre Dame, 2014.

[3] Richard M. Murray, Zexiang Li, and S. Shankar Sastry. *A Mathematical Introduction to Robotic Manipulation*. CRC Press, Boca Raton, FL, first edition, 1994.
