# Supplementary figures and images for: Quantifying the effect of sagittal plane joint angle variability on bipedal fall risk
Source: PLoS One. 2022 Jan 26;17(1):e0262749. doi: 10.1371/journal.pone.0262749 (PMC8791504; doi:10.1371/journal.pone.0262749)

Program flow for simulation of the model SEVEN\_LINK\_AMPUTEE

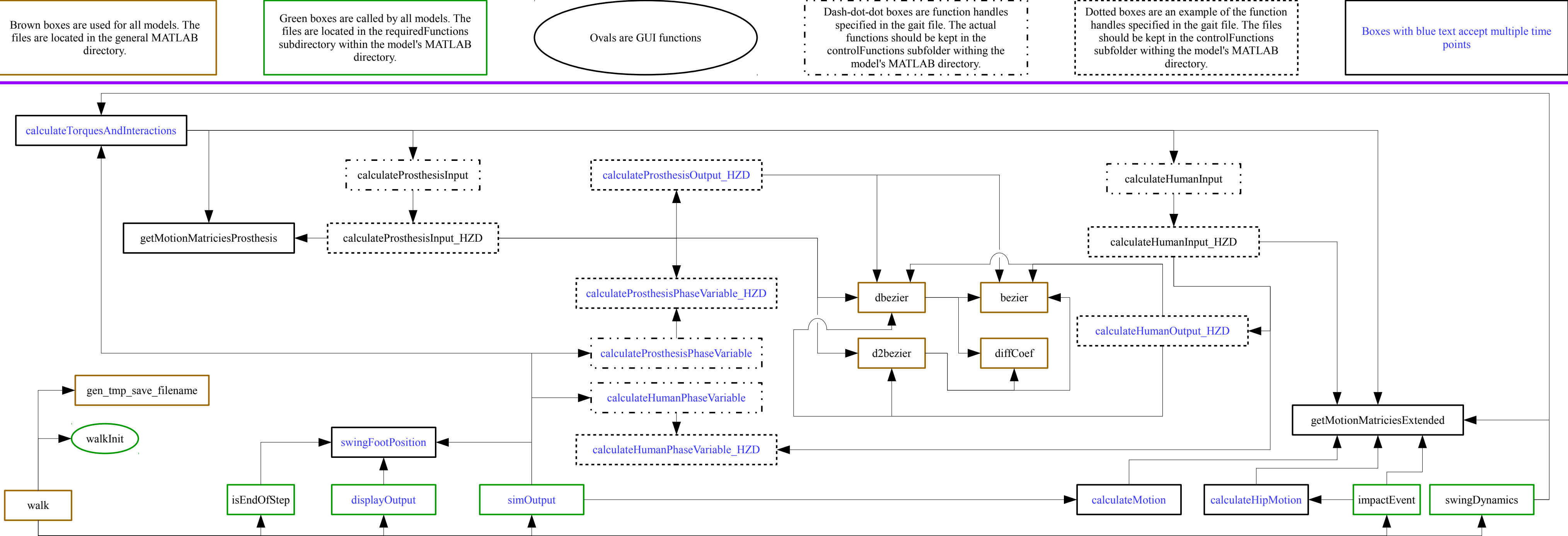

Supplement: S1 File — Matlab code used to generate the simulations. (ZIP) [file pone.0262749.s001.zip › S1_File/RADIUS/models/HEALTHY_HUMAN_REAL_ANKLE/docs/Program Flow.pdf]
